# Local Coordination Motif Compatibility Induces the Formation of Shortened Fe^3+^–O–Ni^3+δ^ Moieties as Active Sites for Highly Efficient Oxygen Evolution Reaction

**DOI:** 10.1021/jacs.6c05060

**Published:** 2026-07-29

**Authors:** Wenchao Wan, Liqun Kang, Shiqian Wei, Alexander Schnegg, Kaltum Abdiaziz, Longxiang Liu, Fei Guo, Christopher S. Allen, Serena DeBeer, Saskia Heumann

**Affiliations:** † 28313 Max Planck Institute for Chemical Energy Conversion, Stiftstraße 34−36, Mülheim an der Ruhr 45470, Germany; ‡ Leshan West Silicon Materials Photovoltaic New Energy Industry Technology Research Institute, Leshan 614000, China; § Department of Chemistry, 4919 University College London, 20 Gordon St, London WC1H 0AJ, United Kingdom; ∥ Electron Physical Science Imaging Centre, 120796 Diamond Light Source Ltd, Didcot, Oxfordshire OX11 0DE, United Kingdom; ⊥ Department of Materials, University of Oxford, Parks Road, Oxford OX1 3HP, United Kingdom

## Abstract

NiFe-based compounds are among the most promising catalysts for the oxygen evolution reaction (OER). However, the structural reconstruction of NiFe catalysts during OER is not fully understood. Most existing studies implicitly assume the formation of a homogeneous NiFe (oxy)­hydroxide lattice; however, the actual reconstruction process is more likely to generate structurally heterogeneous (oxy)­hydroxide phases with local distortions due to the intrinsic mismatch between Fe^3+^–O and Ni^3+^–O bond lengths in the bulk NiFe compounds. By constructing atomically dispersed Ni and Fe active sites as precatalysts and combining them with *operando* spectroelectrochemical studies, we observed an unusual reconstruction pathway in which isolated Ni^2+^ and Fe atoms can adaptively evolve into a short-range mixed NiFe (oxy)­hydroxide local structure through the formation of interconnected M-O-M′ (M/M′ = Ni^3+δ^, Fe^3+^) motifs during the OER. At 1.6 V vs RHE, the reconstructed γ-Fe^3+^OOH clusters are induced to integrate into the high-valent γ-Ni^3+δ^OOH lattice, resulting in a short-range mixed Ni_*x*_Fe_1–x_OOH structure. This new structure is characterized by an unusually short Fe^3+^–Ni^3+δ^ distance of ∼2.86 Å, which is significantly shorter than the typical Fe^3+^-Fe^3+^ distance in Fe^3+^ (oxy)­hydroxides (2.95–3.25 Å). Interestingly, this “induction effect” is absent in Co^3+^–Fe^3+^ catalysts, as atomically dispersed Co^3+^ sites directly transition into γ-Co^3+^OOH, which lacks structural compatibility with γ-Fe^3+^OOH. DFT calculations reveal that the unusually short Fe^3+^-O bond leads to a moderate *O adsorption strength at the Fe site in the catalyst, thereby creating the most favorable conditions for the oxygen evolution reaction (OER).

## Graphical abstract

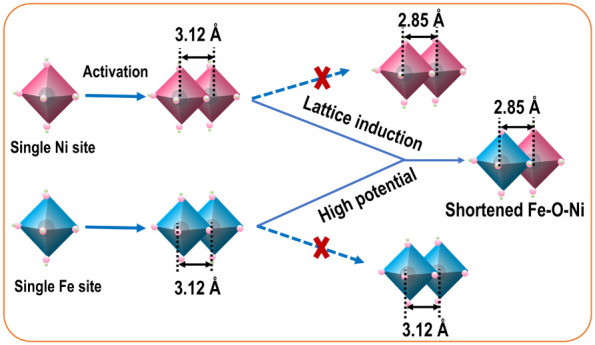

## Introduction

Electrochemical water splitting is pivotal in advancing the transition to a sustainable energy future by enabling renewable hydrogen (H_2_) production, energy storage, and emission-free transportation.^1,2^ The development of earth-abundant electrocatalysts that exhibit exceptional overall water-splitting performance and robust long-term stability is crucial for the practical application of water electrolysis.^3^ The overall efficiency of water electrolysis is largely determined by the sluggish four-electron transfer of the oxygen evolution half-reaction (OER).^4^ Consequently, the urgent development of highly efficient and cost-effective OER electrocatalysts is essential in this field.^5^

In recent decades, significant progress has been made in the design and synthesis of highly active OER catalysts, as well as in understanding the underlying structure-performance relationships and reaction mechanisms.^6^ Earth-abundant and low-cost 3*d* transition metals such as Fe, Co, Ni, compounds are considered among the most promising catalysts, particularly Ni–Fe mixed compounds, which have demonstrated superior OER performance in alkaline electrolytes.^7–9^ In typical NiFe compounds for OER, it is widely accepted that Ni and Fe active centers undergo phase transformations on the surface to form (oxy)­hydroxides.^10^ However, the identification of the true active sites for the OER remains controversial.^11^ For instance, many researchers believed Fe^3+^ species are the active sites for the OER in NiFe catalysts with the Fe content ranging from 10–30% and that the Ni atoms do not contribute to the OER directly but rather serve as a host for Fe incorporation.^12–14^ By contrast, others believe that the catalytic activity arises from the Ni-containing phases,^15^ while Nocera and coworkers suggest that Fe^3+^ promotes the formation of Ni^4+^, which leads to enhanced OER performance.^16^ Sun and coworkers believe that the adjacent Ni and Fe atoms coparticipate in the OER process.^17^ The core of this debate lies in the fact that nearly all OER catalysts undergo structural reconstruction on their surfaces, often resulting in phase separation of Fe and Ni (oxy)­hydroxides or heterogeneous local coordination at the metal centers due to the intrinsic mismatch between Fe–O and Ni–O bond lengths.^18–20^ However, the insufficient clarity regarding the reconstruction dynamics and the ambiguity in determining the actual catalytically active species present substantial obstacles to deciphering the underlying mechanisms.

Despite the ongoing controversy, a consensus has emerged that the high oxidation state of Ni species, as well as the synergistic effect of Fe and Ni atoms, plays an indispensable role in enhancing OER performance.^21,22^ It is well established that the formation of high-valent metal species (primarily Ni^3+/4+^) with a shorter distance of Ni–Ni (around 2.84 Å) is crucial for the OER, while the Fe sites are usually very stable.^23^ This may be related to the fact that the oxidation state of Fe remains predominantly as Fe^3+^ in most cases.^24,25^ Usually, Fe and Ni each form their respective (oxy)­hydroxide phases during OER. Due to the differences in bond lengths between the Ni and Fe (oxy)­hydroxide phases—for example, the bond length of Ni^2+/3+^–O usually ranges from 1.86 to 2.08 Å,^26,27^ significantly shorter than the bond length of Fe^3+^-O which usually ranges from 1.91 to 2.15 Å in different (oxy)­hydroxides.^14^ When Ni is in its high-valence state (Ni^3+^OOH), the difference can be as large as 0.2 Å. These two metal centers are likely to form separate phases with different coordination environments during surface structural reconstruction.^25,28^ In other words, only a small portion of Ni and Fe atoms form Ni^3+^–O–Fe^3+^ moieties (most likely at the boundary of the two phases) within the newly formed active phase. However, this has been overlooked in previous studies, which continue to use a uniform Ni_*x*_Fe_1–*x*_OOH phase as structural models for reaction mechanism investigations.^29,30^ In 2015, for the first time, using *operando* X-ray absorption spectroscopy (XAS), the formation of an unusually short Fe^3+^–O bond length (1.90 Å) with a similar lattice structure with edge-sharing high-valent γ-Ni^3+^OOH was observed.^14^ This study represents one of the few observations of extremely short Fe^3+^–O bonds during the water oxidation process.^31^ A recent simulation study reveals that such an atypically short Fe^3+^–O bond length is typically formed when the Fe:Ni ratio is maintained below 25% in the (oxy)­hydroxides.^32^ This may stem from the inherent limitations of the typically used sol–gel method for (oxy)­hydroxides, where achieving atomical-level control over the formation of Ni^3+^–O-Fe^3+^ moieties is challenging. Notably, the experimental origin of this anomalous bond shortening remains unresolved, and the interactions between Ni and Fe atoms during surface reconstruction, as well as the formation of the ultimate catalytically active species, are still unclear.

In order to study the structural reconstruction of Ni and Fe sites and their actual contribution to the OER, in this study, we utilized a supramolecular coordination strategy to synthesize atomically dispersed NiFe (mass ratio of Fe:Ni ≈ 1:2) precatalyst for OER. During the activation process, the catalyst gradually converted into a short-range mixed NiFe oxyhydroxide local structure with efficient OER performance. *Operando* XAS studies indicate that atomic dispersion promotes Ni and Fe atoms to first form their respective hydroxide-like species, such as Ni­(OH)_2_ and γ-FeOOH, during the electrochemical activation process. Due to the similarity of the structure, the distance of Fe^3+^–Fe^3+^ in γ-FeOOH is around 3.12 Å, which is similar to that of Ni^2+^-Ni^2+^ in Ni­(OH)_2_. These two species show strong structural compatibility during formation and were well integrated. Upon increasing the voltage to 1.6 V vs RHE, Ni^2+^ was oxidized into high-valent Ni^3+δ^, resulting in the formation of short-range mixed Ni_*x*_Fe_1–*x*_OOH clusters featuring an unusually short Fe–O–Ni distance of ∼2.86 Å. Comparative analysis with individual Fe-only and Ni-only catalysts demonstrates that, in the absence of Fe atoms, Ni atoms can still form Ni­(OH)_2_ at low voltages (<1.4 V) and transition to γ-Ni^3+δ^OOH-like phase at higher voltages. However, structures containing only Fe atoms did not exhibit this shortened Fe^3+^-O bonding. This provides key evidence that the formation of this short-range mixed Ni_*x*_Fe_1–*x*_OOH-like phase with unusually short Fe^3+^–O bonds needs the presence of Ni^2+^ atoms. Interestingly, this induction effect was not observed when replacing Ni^2+^ with Co^3+^ in the precatalysts, as the Co^3+^ sites were directly converted into a γ-CoOOH-like phase (with Co^3+^-O bond length and Co^3+^–Co^3+^ distance of 1.90 Å and 2.86 Å, respectively) from the outset, which is not geometrically compatible with the structure of Fe^3+^OOH (Fe^3+^–O bond length and Fe^3+^–Fe^3+^ distance of ∼2.00 Å and ∼3.09 Å), thereby precluding the induction effect. Density functional theory (DFT) calculations further reveal that the electron deficiency of high-valent Ni^3+δ^ sites induces a substantial positive charge density, which is subsequently transmitted to Fe^3+^ via metal–oxygen bridges (Ni–O–Fe). This charge transfer lowers the electron density around Fe^3+^, significantly reducing the energy barrier for the *O to *OOH transition and thereby enhancing the catalytic activity of Fe sites for OER.

## Results and Discussion

### Catalysts Syntheses and Characterizations

Inspired by supramolecular metal–organic chemistry, natural polyphenol tannic acid (TA) was selected as a ligand to coordinate Fe^3+^ and Ni^2+^ ions through the regulation of pH in water. Meanwhile, oxygen-functionalized CNTs were used as a support to stabilize the TA complexes (Figure 1a; see details in Supplementary). The selection of CNTs is due to their high electron-transfer ability and their capacity to stabilize TA molecules via noncovalent π–π interactions and van der Waals forces.^33^ Tannic acid ligands and the functionalized CNT surface likely work synergistically to stabilize atomically dispersed Ni and Fe species and confine the reconstructed ultrathin NiFe oxyhydroxide domains. The phenolic hydroxyl groups of tannic acid can coordinate Ni and Fe in the precursor state and may further interact with coordinatively unsaturated metal sites during reconstruction. Meanwhile, oxygen-containing functional groups on the CNT surface can serve as anchoring sites for both the ligands and reconstructed oxyhydroxide species. These interactions help suppress excessive particle growth, long-range metal migration, and phase segregation during OER-induced reconstruction.

**Figure 1:**
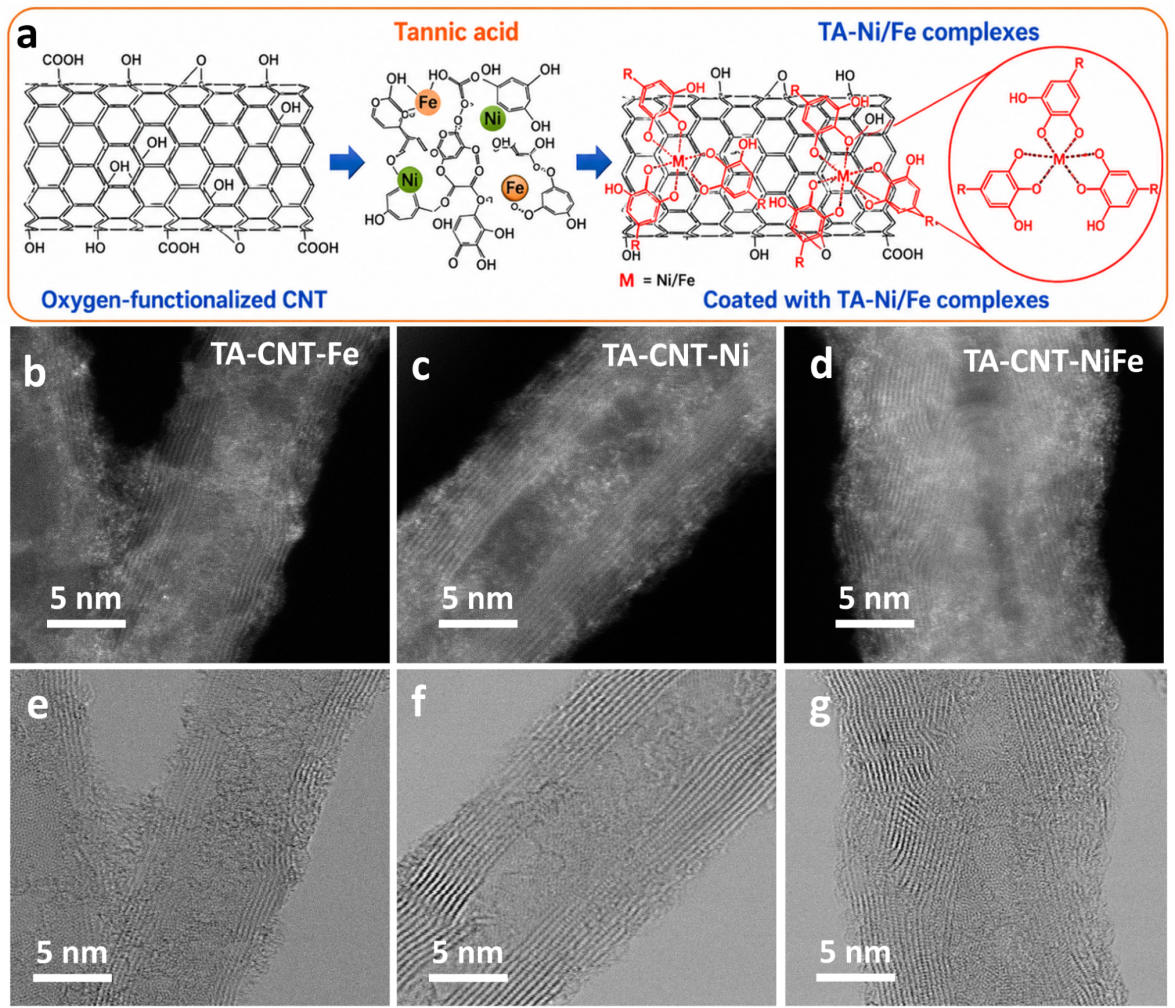
(a) Schematic diagram for the synthesis of a six-coordinated single active site NiFe OER catalyst on CNTs. HAADF-STEM images of (b) TA-CNT-Fe, (c) TA-CNT-Ni, (d) TA-CNT-NiFe, and (e–f) their corresponding BF-STEM images.

The atomic dispersion of metal centers and ultrathin layers was confirmed directly by high-angle annular dark field scanning transmission electron microscopy (HAADF-STEM) (Figure 1b–g). In these images, due to the power-law dependence of HAADF-STEM intensity on atomic number, heavier atomic species appear brighter. Atomically dispersed Fe and Ni are visualized as distinct bright dots on CNTs. The metal contents of TA-CNT-Fe, TA-CNT-Ni, and TA-CNT-NiFe are 0.83, 1.50, and 1.27 wt % (Fe: 0.46 wt %, Ni: 0.81 wt %), respectively, as acquired by X-ray fluorescence (XRF) measurements (Table S1). The Fe content in this work lies within the reported Fe solubility window of NiOOH (∼25–35%), above which Fe-rich segregation is thermodynamically favored, likely due to spin-state changes and local structural mismatch.^25,32^ Raman spectroscopy and powder X-ray diffraction (PXRD) were employed to investigate the structure of the catalysts (Figure S1). Raman spectra indicate that the catalysts contain significant CNT signals, with typical D, G, and 2D bands at around 1349 cm^–1^, 1586 cm^–1^, and 2695 cm^–1^, respectively. In PXRD patterns, only signals from CNT and TA were observed, indicating the amorphous feature of TA-coordinated metal complexes.

In order to assess the electronic structure and local environment of the atomic metal centers, the Ni and Fe K-edge absorption near-edge structure (XANES) spectra were performed. At the Ni K-edge, the absorption edge positions for TA-CNT-Ni and TA-CNT-NiFe are close to that of Ni­(OH)_2_, suggesting the domination of Ni^2+^ in the catalysts (Figure 2a). Meanwhile, the pre-edge peak position of TA-CNT-Ni and TA-CNT-NiFe coincides with that of Ni­(OH)_2_, and is clearly shifted to lower energy with respect to γ-NiOOH. Such a narrow, moderately intense pre-edge is also characteristic of Ni^2+^O_6_ octahedra with slight deviation from a centrosymmetric structure, as well as evidence of the absence of high-spin Ni^3+^/Ni^3+δ^ species (Figure 2b).^34^

**Figure 2:**
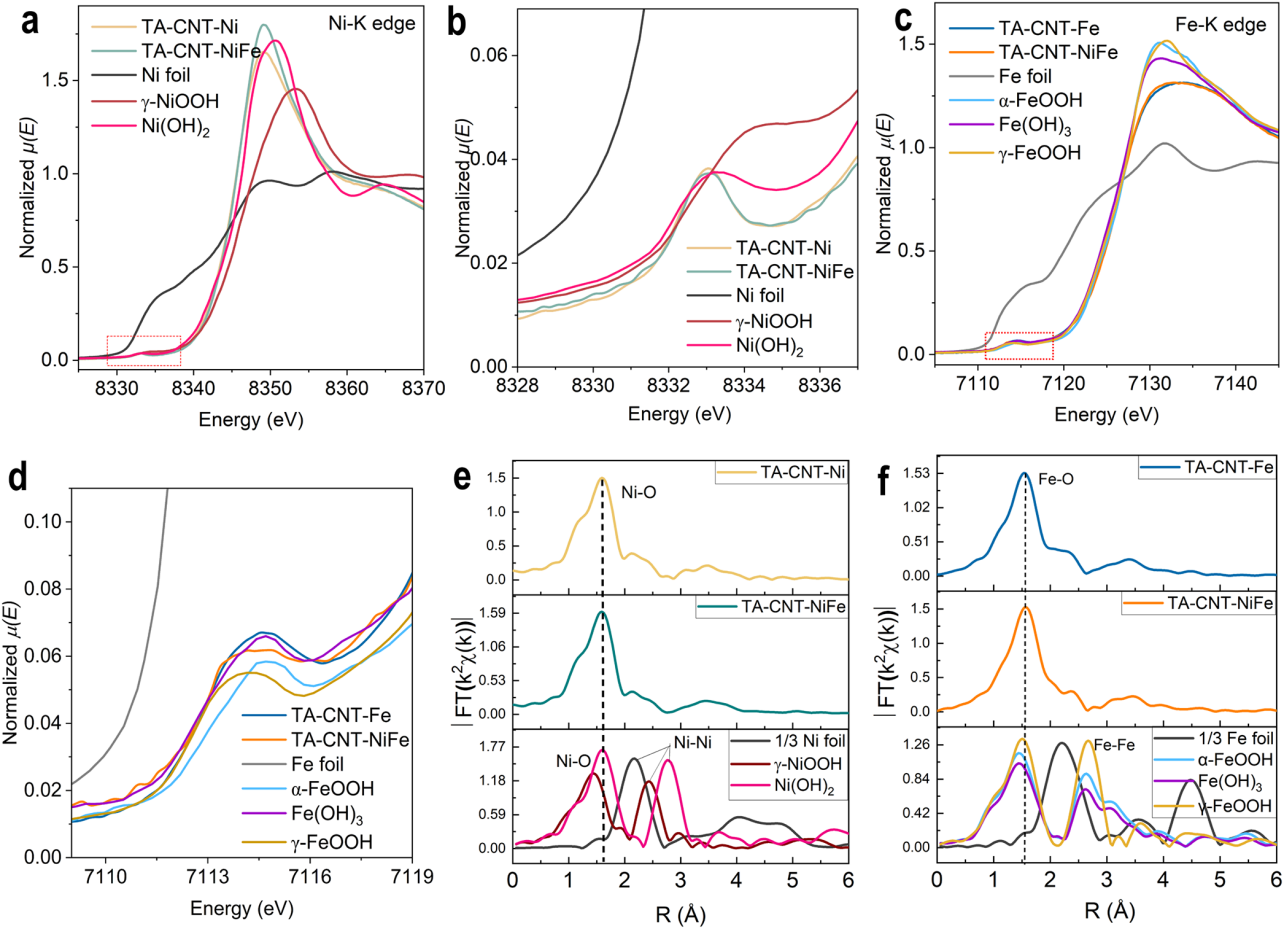
(a–d) K-edge XANES and pre-edge spectra of TA-CNT-Ni, TA-CNT-Fe, TA-CNT-NiFe, and their reference samples. (e–f) Fourier transform of K-edge EXAFS spectra of TA-CNT-Ni, TA-CNT-Fe, TA-CNT-NiFe, and their reference samples.

At the Fe K-edge, the absorption edge positions for both TA-CNT-Fe and TA-CNT-NiFe overlap with Fe­(OH)_3_ and α/γ-FeOOH, which indicates that the average valence state of Fe species is Fe^3+^ (Figure 2c). The pre-edge region of TA-CNT-Fe and TA-CNT-NiFe is characterized by a broad feature at ∼7114 eV with intensity comparable to that of α/γ-FeOOH. Such pre-edge shape and intensity are typical of high-spin Fe^3+^O_6_ octahedra with moderate distortion in Fe (oxy)­hydroxides (Figure 2d).^35^ The Fourier-transform extended X-ray absorption fine structure (FT-EXAFS) spectra were further collected for all as-prepared catalysts. The single peak located at around 1.55 Å (without phase correction) for all catalysts indicates atomic dispersion of the metal species (Figure 2e–f). This is further evident from the analyses of their wavelet transform (WT)-EXAFS spectra (Figure S2), where only one scattering pathway at the lower k-range is observed for all catalysts. The bond length of Ni–O in TA-CNT-Ni and TA-CNT-NiFe is closer to that of Ni^2+^ coordinated with OH^–^ in Ni­(OH)_2_, and the CNs of Ni centers in TA-CNT-Ni and TA-CNT-NiFe are 6.2 and 6.2, as obtained from the fitting results (Table S2). The bond lengths of Fe–O in TA-CNT-Fe and TA-CNT-NiFe are nearly identical and are similar to that of Fe–O in γ-FeOOH (∼2.0 Å). The fitting results show that the coordination numbers (CNs) of Fe centers for TA-CNT-Fe and TA-CNT-NiFe are 6.0 and 6.4, respectively (Table S2). This is in line with the six-coordinated octahedral geometry as concluded in the analyses of XANES (Figure 2a, c).

The structural information is further confirmed by the EPR spectra. The TA-CNT-M (M = Fe, Co, Ni, CoFe, and NiFe) as prepared are shown in Figure S3. TA-CNTs-Co exhibits a broad feature at *g*_eff_ = 5.2, characteristic of high-spin Co^2+^ (S = 3/2) in octahedral coordination.^36^ TA-CNTs-Fe displays a sharp signal at *g*_eff_ = 4.2 with a shoulder at *g*_eff_ = 9 and a broad component at *g*_eff_ = 2. Similar EPR spectra have been reported for iron oxide nano particles and Fe-doped layered hydroxides and have been assigned to five- and six-coordinate high-spin (HS, S = 5/2) Fe^3+^.^37,38^ The narrow signal at *g*_eff_ = 4.2 was assigned to magnetically isolated or weakly coupled HS Fe^3+^ and the broad component to strongly magnetically coupled HS Fe^3+^. It was shown for Fe-doped layered hydroxides that, upon increasing the iron content and thereby the magnetic coupling between the iron sites, the spectrum transitions from the broad spectrum around *g*_eff_ = 2 to the rhombic *g*_eff_ = 4.2 spectrum. Since, in our case, mainly the latter component is observed, only weak magnetic interaction between the Fe sites is assumed.^37^ TA-CNTs-Ni (Figure S3) shows a broad signal centered at *g*_eff_ = 2.1 with a line width of ∼100 mT, indicative of strongly magnetically coupled clustered Ni species. Parallel-mode EPR, used in previous work to probe high-spin Ni^2+^ (*S* = 1) in Ni-doped layered hydroxides, showed no EPR signal; however, this may be due to the lower detection sensitivity in parallel-mode EPR.^37^ The bimetallic TA-CNTs-CoFe (Figure S3) and TA-CNTs-NiFe (Figure S3c) resemble a superposition of the corresponding monometallic TA-CNTs. This indicates that the magnetic coupling between the transition metal sites does not significantly increase in the mixed systems, as one would expect, for e.g., for oxygen-bridged paramagnetic Co–Fe sites.

### Electrochemical OER Characterizations

The as-prepared catalysts were assembled as anodic electrodes and tested in 1.0 M KOH for OER. All catalysts were first activated. It was found that the current densities of the first CV scan for all catalysts were much larger than the subsequent scans. The second and the 20th CV cycles were quite similar, implying the occurrence of potential structural reconstruction during the first CV cycle. Compared to monometallic catalysts, TA-CNT-NiFe exhibits excellent OER performance. A pronounced redox peak was observed in the CV test of TA-CNT-Ni, which can be assigned to the transition of Ni^2+^ to Ni^3+^(Figure 3a). The cathodic Ni^2+^/Ni^3+^ redox peak of TA-CNT-Ni was integrated to estimate the amount of electrochemically addressable Ni sites. Assuming a one-electron Ni^2+^/Ni^3+^ process, the 20th-cycle redox charge of 17.86 mC corresponds to 0.185 μmol of Ni redox sites, close to the total Ni amount of 0.192 μmol estimated from the catalyst loading and Ni content; the detailed fitting is provided in Table S5. This suggests that most Ni sites in TA-CNT-Ni were converted into the NiOOH phase. A redox feature was also observed for TA-CNT-NiFe during CV activation, but it was markedly attenuated and shifted to more positive potentials compared with TA-CNT-Ni. We did not convert the integrated redox charge into an absolute NiOOH amount because Fe incorporation can perturb the Ni-centered redox process and introduce overlapping contributions from Ni–Fe oxyhydroxide species. Thus, this broad redox response cannot be treated simply as a one-electron Ni^2+^/Ni^3+^ transition. Instead, the positive shift and stabilization of the redox feature are interpreted as qualitative evidence for Fe-induced reconstruction of a NiOOH-like lattice. Such Fe-induced modulation of Ni redox behavior is widely observed in NiFe oxyhydroxide catalysts and is generally associated with changes in the local electronic structure of Ni sites, including redistribution of Ni d-orbital electron density upon Fe incorporation. Since the OER activity of Ni-based oxyhydroxides is closely related to the electronic structure of Ni d states, Fe incorporation can alter both the position and intensity of the Ni redox peaks.^39,40^ Notably, analogous redox features have been reported for NiFe systems containing well-defined Ni–O–Fe motifs. Fe incorporation into NiOOH has been shown to generate Fe–O–Ni moieties, thereby enhancing OER activity and improving the stability of Ni-based oxyhydroxide catalysts.^41^ Moreover, a positive shift of the Ni oxidation peak accompanied by attenuation of the corresponding reduction peak has been identified as an electrochemical signature of Fe incorporation into the NiOOH lattice and the formation of Ni–Fe oxyhydroxide motifs.^42^ In our system, these features appear after the first CV cycle, suggesting that the initially dispersed Ni and Fe sites undergo electrochemical reorganization to form reconstructed NiFe oxyhydroxide species.

**Figure 3:**
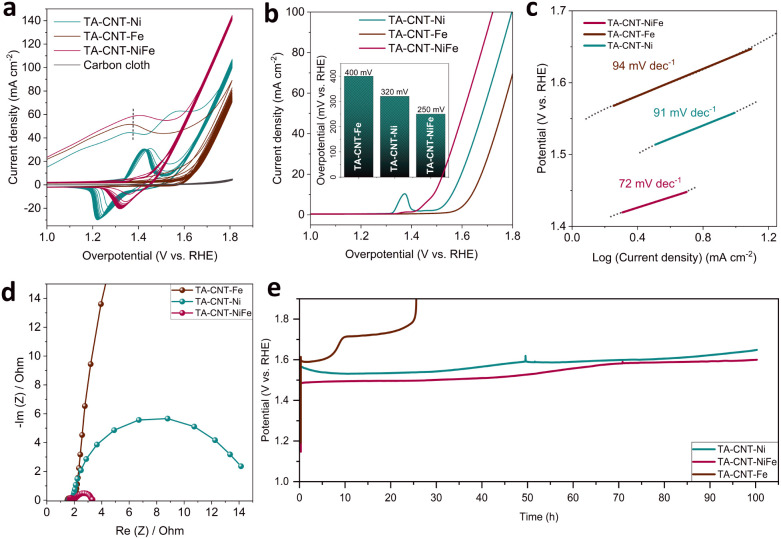
Electrocatalytic OER performances of the as-investigated catalysts in 1 M KOH for the OER. (a) CV curves, (b) LSV curves, (c) Tafel slopes, (d) Nyquist plots (at 1.50 V vs RHE), and (e) chronopotentiometry measurements.

The LSV test also shows that TA-CNT-NiFe is significantly more active than monometallic catalysts and recently reported NiFe catalysts in the literature, with an overpotential of only 250 mV (vs RHE) at 10 mA cm^–2^ without IR compensation (Figure 3b, Table S6). This is also observed in the mass activity of the catalysts, indicating that the enhanced OER activity in TA-CNT-NiFe is not related to the metal content but to the synergistic effect of Ni and Fe (Figure S4). Tafel plots and electrochemical impedance spectroscopy (EIS) measurements were also conducted to shed light on the intrinsic OER kinetics of the investigated catalysts (Figure 3c). A Tafel slope value of 72 mV dec^–1^ is observed in TA-CNT-NiFe, which outperformed those of TA-CNT-Ni and TA-CNT-Fe, indicating facilitated OER kinetics with the combination of Ni and Fe atoms. The EIS results show that the charge transfer resistance (R_ct_) in TA-CNT-NiFe is significantly smaller than those in TA-CNT-Ni and TA-CNT-Fe (Figure 3d). The TA-CNT-NiFe and TA-CNT-Ni catalysts also exhibit excellent stability, showing no significant performance degradation after 100 h (Figure 3e). The slight performance decay observed at the later stage of the test may be associated with partial structural relaxation, minor phase segregation, or other gradual degradation processes. Nevertheless, this decay is not severe, and the overall curve remains relatively stable, indicating that rapid irreversible structural collapse or extensive phase separation is unlikely under operating conditions. ICP-MS analysis of the electrolyte before and after the stability test indicates that the concentrations of metal species in solution remain essentially unchanged (Table S7). Therefore, the change in metal leaching can be considered negligible. *Operando* EIS tests were further conducted to investigate the role of the synergetic effect of Fe and Ni during the OER (Figure S5). As the applied potential increases, the radii of the EIS spectra for all catalysts decrease dramatically, indicating that the electrochemical processes on the electrode surface evolve at different voltages. The Bode plots further confirm changes in the surface state at various potentials. A strong signal emerges in the low-frequency region (<10 Hz), typically representing the ion diffusion process at the interface. This process is significantly suppressed as the potential increases, while the signal in the midfrequency region (10 Hz to 1 kHz), which corresponds to the charge transfer process, becomes dominant. This also indicates the catalytic OER is triggered at this moment. Notably, the potential required to trigger the OER for the TA-CNT-NiFe (after 1.4 V vs RHE) is much lower than that for TA-CNT-Ni (after 1.5 V vs RHE) and TA-CNT-Fe (after 1.6 V vs RHE). It is worth noting that the onset potential of each catalyst is expected to be lower than the voltages mentioned above. The high-frequency region (1 kHz to 1 MHz) generally reflects the resistance of the electrolyte, and at higher potential, this process becomes dominant in all catalysts (Figure S5). Electrochemical analysis shows that combining Fe and Ni single sites significantly improves OER kinetics and performance compared to monometallic catalysts. This indicates that during the OER, Fe and Ni undergo structural rearrangement, forming synergetic active sites similar to those in other widely reported NiFe compounds.

### Dynamics of Phase Transition Derived from In Situ XAS

To further confirm the structural rearrangement and newly formed active sites during the OER process, *operando* XAS measurements were conducted (Figure S6). In the Ni K-edge XANES of TA-CNT-NiFe, the absorption energy increases with applied potential, from the open circuit voltage (OCP, after 20 cycle CVs) to 1.6 V vs RHE, indicating an increase in the oxidation state of Ni species in the catalyst (Figure 4a). The absorption edge shifts by 2.5 eV when the potential increases from OCP to 1.6 V vs RHE (Figure 4b–c). Initially, the oxidation state of Ni species in the catalysts is close to β-Ni^2+^(OH)_2_, with a corresponding absorption edge energy at around 8343.0–8343.5 eV, whereas the absorption edge position of γ-Ni^3+^OOH is approximately 8344.5 eV. Such a significant edge shift of ∼2.5 eV is significantly larger than the ∼1.3 eV edge shift typically observed between β-Ni^2+^(OH)_2_ and γ-Ni^3+^OOH, indicating that at 1.6 V, the Ni centers in TA-CNT-NiFe are oxidized beyond the level of conventional γ-Ni^3+^OOH. The presence of such highly oxidized Ni^3+δ^ species under OER conditions is in line with previous observations in Fe–Ni OER catalysts.^14,25–46^ The pre-edges of the samples at different potentials also follow the same trend (Figure 4d). The position and intensity of the pre-edges initially overlap with those of Ni^2+^(OH)_2_. However, at 1.6 V vs RHE, the pre-edge moves toward the characteristic of γ-Ni^3+^OOH, but with a noticeably greater intensity. This further indicates that while the oxidation state is comparable to γ-Ni^3+^OOH, the Ni centers adopt a different coordination geometry (Figure 4d). This may arise from the Ni species existing as highly amorphous clusters rather than adopting the ordered structure of crystalline γ-Ni^3+^OOH. The shift of the absorption energy and transition of the pre-edge for TA-CNT-Ni during OER are very similar to TA-CNT-NiFe (Figure S7); however, the Ni^2+^ to Ni^3+^/Ni^3+δ^ oxidation started at a much lower applied potential (1.4 V vs RHE). This indicates that structural reconstruction happened much easier without the presence of Fe.

**Figure 4:**
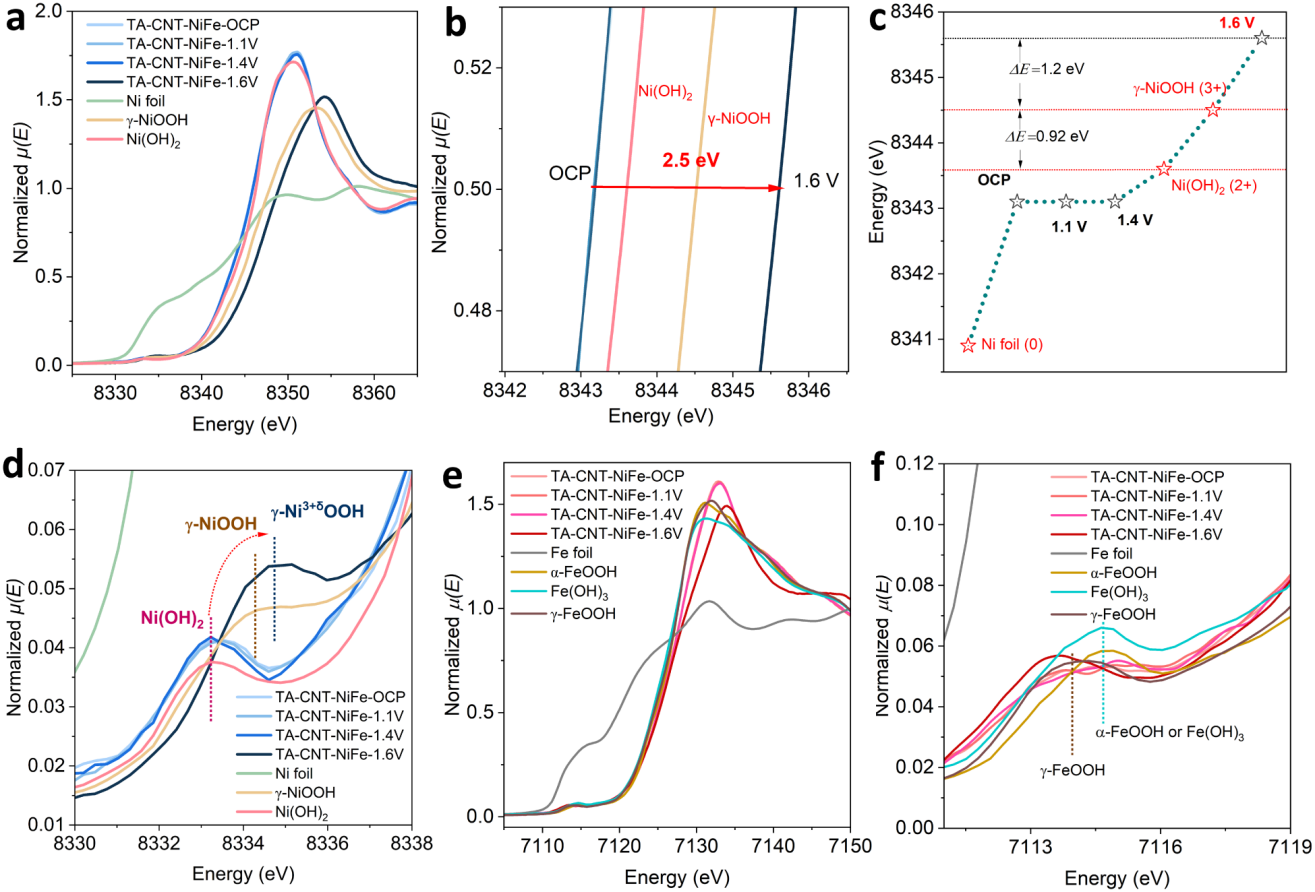
(a, e) *Operando* XANES spectra of TA-CNT-NiFe collected at different potentials and the reference compounds. (b–c) Plot of the shift in the absorption energies of XANES at different potentials and comparison with similar reference compounds. (d, f) Ni and Fe K-pre-edge spectra of TA-CNT-NiFe at different potentials and comparison with similar reference compounds.

At the Fe K-edge, the XANES position of the TA-CNT-NiFe catalyst remains essentially unchanged from OCP to 1.6 V (Figure 4e), and the pre-edge features preserve both their energy and shape across the entire potential range (Figure 4f). This indicates that the formal oxidation state of Fe species stays predominantly at Fe^3+^. Notably, however, between 1.4 and 1.6 V, the white-line region undergoes a clear evolution: its intensity decreases, and its maximum shifts to slightly higher energy. Such changes are typically associated with modulations in the local coordination environment rather than changes in oxidation state and are consistent with a restructuring of the first coordination shell, for example, through bond length contraction or altered octahedral distortion.^31,47^ Considering the simultaneous Fe–O bond contraction observed by EXAFS, this white-line evolution is more likely associated with compressed/distorted FeO_6_ coordination and enhanced Fe–O–Ni covalency induced by the oxidation/deprotonation of the surrounding Ni oxyhydroxide network. Similar interpretations have been reported for Fe-doped Ni oxyhydroxides, where operando XANES analysis and simulations associated changes in white-line intensity and edge features with FeO_6_ distortion, deprotonation-induced contraction, and enhanced Fe–O covalency within γ-NiOOH-like environments.^31,48^ Specifically, the Fe-site transformation in TA-CNT-NiFe does not occur independently but is coupled to the oxidation, deprotonation, and contraction of the neighboring Ni hydroxide/oxyhydroxide framework. As Ni^2+^-like species are oxidized toward Ni^3+^/Ni^3+δ^ oxyhydroxide under anodic bias, the contraction of the surrounding Ni–O network can perturb the electronic structure of bridging oxygen atoms, thereby modifying Fe–O–Ni covalency and making the FeO_6_ environment more contracted and electronically perturbed without requiring the formation of a dominant Fe^4+^ population. In contrast, the TA-CNT-Fe catalyst exhibits XANES and pre-edge spectra that are nearly identical across all applied potentials (Figure S8). In this case, the Fe sites undergo negligible structural evolution during OER. This comparison suggests that the Fe-site evolution in TA-CNT-NiFe is not simply caused by anodic polarization of isolated Fe species but is closely related to the evolving Ni oxyhydroxide matrix. It is also noteworthy that the pre-edge and white-line shapes of TA-CNT-Fe and TA-CNT-FeNi differ from each other and from those of bulk α/γ-FeOOH. This deviation is expected from nanoscale Fe environments, where reduced symmetry and structural disorder can lead to electronic structures distinct from crystalline reference phases. Notably, even at OCP, the Fe K-edge white-line feature of TA-CNT-NiFe is already distinct from that of TA-CNT-Fe, suggesting that Fe is incorporated into a Ni hydroxide-like local environment rather than remaining in an FeOOH-like or phase-separated environment. Overall, these observations confirm that although Fe is dominated with Fe^3+^ oxidation state in both systems, the Fe coordination environment in TA-CNT-NiFe evolves noticeably from 1.4 to 1.6 V, while that in TA-CNT-Fe remains essentially invariant. The absence of a clear positive shift in the absorption edge or pre-edge centroid further argues against dominant Fe^3+^→Fe^4+^ oxidation; instead, the spectral evolution is better assigned to changes in Fe–O bond length, local distortion, and Fe–O–Ni covalency. From a spectroscopic perspective, the Fe K-edge white line is sensitive not only to oxidation state but also to Fe–O bond length, coordination geometry, metal–ligand covalency, and second-sphere effects transmitted through bridging oxygen atoms. Therefore, the decreased white-line intensity, slight white-line shift, and pre-edge evolution under OER bias are more reasonably attributed to compressed/distorted FeO_6_ coordination and modified Fe–O–Ni covalency within the evolving Ni oxyhydroxide environment. The pre-edge evolution further supports this interpretation. The Fe K-edge pre-edge mainly originates from the 1*s*→3*d* transition and is highly sensitive to local symmetry, Fe-ligand covalency, ligand-field splitting, and 3*d*-4*p* mixing, rather than oxidation state alone.^49–51^ Therefore, the observed pre-edge change is more likely associated with local geometric distortion and modified Fe–O–Ni covalency. In particular, Ni oxidation and Ni–O framework contraction can alter the electronic structure of bridging oxygen atoms and Fe 3*d*–O 2*p* antibonding orbitals, while FeO_6_ compression/distortion can modify *d*-orbital splitting and the relative contributions of different pre-edge components. These effects can change the pre-edge feature without requiring dominant Fe^4+^ formation. Nevertheless, a minor high-valent Fe (such as Fe^4+^) contribution cannot be completely excluded because XANES is an ensemble-averaged local structural probe.

Ni and Fe K-edge Fourier transform (FT) EXAFS was further employed to confirm the evolution of local coordination (Figure 5a–b). From the Ni K-edge FT-EXAFS, at the applied potential of OCP, the fitted C.N. of the first coordination shell Ni–O is 5.9 (2.06 Å), which is identical to that of Ni­(OH)_2_ (Table S3, Figures S26–59). In contrast to the fresh catalyst, a strong second-shell coordination at 3.09 Å was identified, attributed to the Ni–Ni/Fe scatterings with a C.N. of 5.9. This unambiguously suggests the aggregation of isolated Ni single sites and the formation of nanostructures similar to Ni­(OH)_2_ after 20 cycle CVs. As the potential continues to rise from 1.4 to 1.6 V, the Ni–O bond length and Ni–Ni/Fe distance reduced significantly to 1.89 Å and 2.82 Å, respectively, which are similar to but even slightly shorter than the corresponding distances observed in crystalline γ-Ni^3+^OOH. Therefore, one can infer that the Ni­(OH)_2_ phase further transforms into γ-Ni^3+δ^OOH together with increase of the oxidation state. This agrees well with the above discussion about the pre-edge of Ni species. Ni­(OH)_2_ was completely converted into high-valent γ-Ni^3+δ^ OOH with edge-sharing Ni^3+δ^ O_6_ octahedra (Figure 4d). Consistent with the Ni K-edge XANES behavior, the FT-EXAFS analysis of TA-CNT-Ni shows an overall evolution trend similar to that of TA-CNT-NiFe, with the primary difference being the voltage at which the structural transition occurs (Figure S9). In the absence of Fe, the Ni­(OH)_2_ to γ-Ni^3+δ^OOH phase transition starts at a significantly lower potential. At around 1.4 V, the EXAFS fitting reveals the coexistence of Ni­(OH)_2_ and γ-Ni^3+δ^OOH-like coordination structures, indicating that partial oxidation has already taken place. Upon increasing the potential to 1.6 V, the transformation becomes complete, and the spectrum is consistent with the high-valent γ-Ni^3+δ^OOH phase (Figure S10, Table S3, Figures S26–59). This lower onset potential of the Ni oxidation process aligns well with the CV measurements, where the Ni^2+^/Ni^3+^ redox peak appears at approximately 1.35 V (vs RHE) for TA-CNT-Ni compared to the higher potential of 1.45 V (vs RHE) observed for TA-CNT-NiFe (Figure 4a).

**Figure 5:**
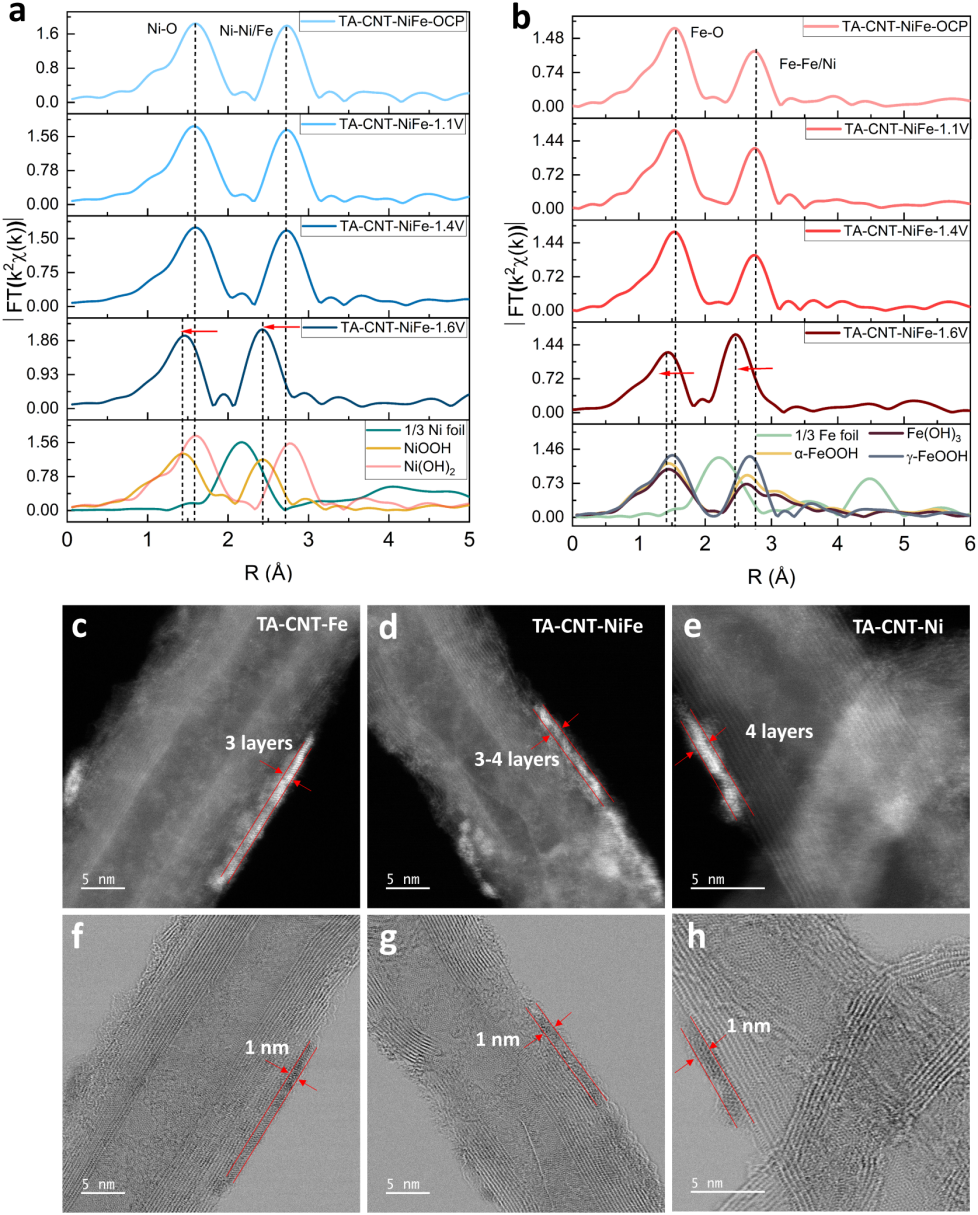
Experimental Ni and Fe K-edge (a and b) Fourier transform EXAFS signals of TA-CNT-NiFe. HAADF-STEM images of (c, f) TA-CNT-Fe, (d, g) TA-CNT-NiFe, and (e, h) TA-CNT-Ni after *operando* XAS measurements.

The Fe K-edge FT-EXAFS spectra of TA-CNT-NiFe reveal several characteristic changes during the OER activation. The first-shell Fe–O coordination (∼2.00 Å) remains unchanged from the initial state, with a coordination number close to 6, indicating that the Fe sites retain the octahedral Fe^3+^O_6_ environment throughout. After 20 CV cycles (OCP), the second-shell scattering path Fe–Ni/Fe is also detected at around 3.12 Å, consistent with the observation of (oxy)­hydroxide clustering in Ni K-edge EXAFS. These EXAFS coordination features remain largely unchanged until the potential reaches 1.6 V, where all fitted Fe–O and Fe–Ni/Fe distances contract noticeably to 1.91 Å and 2.86 Å, respectively. The assignment of the 2.86 Å distance to Fe–Ni/Fe rather than Fe–C/N/O scattering is further supported by the wavelet transform analysis of the *k* ^2^-weighted EXAFS signal. As shown in Figure S11, the pronounced scattering feature at relatively high *k* values is characteristic of metal–metal scattering paths, rather than light-atom scatterers such as C, N, or O. Such shortening could superficially be interpreted as arising from Fe oxidation; however, the Fe K-edge XANES shows no meaningful edge shift, and pre-edge features remain characteristic of Fe^3+^. Therefore, the observed bond-length contraction is more reasonably attributed to a restructuring of the Fe coordination environment rather than the formation of even higher valence Fe species. This interpretation is also fully consistent with the subtle white line intensity and energy changes observed in the corresponding XANES spectra. The Fe-only reference catalyst TA-CNT-Fe shows nearly identical FT-EXAFS spectra across all applied potentials. Both the Fe–O and Fe–Fe coordination shells remain unchanged and match well with those expected for FeOOH structures (Figure S10, Table S8), confirming that Fe centers alone do not undergo significant structural reorganization during OER.

The formation of (oxy)­hydroxide is also evident from the HAADF-STEM measurement, where ultrathin nanosheets with about 3–4 layers (∼1 nm) and sizes ranging from a few to 20 nm were observed (Figure 5c–h). We also applied FFT image processing together with feature extraction and identification to further verify the crystallographic facets of the clusters, comparing them with standard (oxy)­hydroxide diffraction references (Figures S60–83, Table S9). The results show that the Fe, Co, and Ni species all form (oxy)­hydroxide-like clusters. The Raman spectra obtained after the OER further confirmed the formation of M-O-M species (Figures S12–S14, Table S10). In addition to the characteristic signals of CNTs, namely the D, G, and 2D bands, distinct M-O stretching vibrations were observed at approximately 600–680 cm^–1^ for TA-CNT-Ni, TA-CNT-Fe, and TA-CNT-NiFe.^22^ Notably, catalysts containing Ni atoms also exhibited a peak around 1060 cm^–1^, which corresponds to the characteristic MOO^–^ species, consistent with previous reports.^52,53^

### Formation Mechanism of Short Fe^3+^–O–Ni^3+δ^ Moieties

Several independent observations demonstrate that the formation of shortened Fe–O–Ni linkages in TA-CNT-NiFe arises specifically from the cooperative interplay between Fe and Ni. First, the oxidation of Ni is shifted to higher potentials in the presence of Fe, as revealed by both Ni K-edge XANES and CV, indicating that Fe perturbs the electronic structure and reconstruction pathway of Ni sites during activation.^46^ Second, *operando* EXAFS shows that upon increasing the potential to 1.6 V, both metals undergo a simultaneous contraction of their M-O and M-M coordination distances, and the fitted Fe-M and Ni-M distances converge to nearly the same value (approximately 2.86 Å). This synchronous structural response is incompatible with the independent formation of separate γ-NiOOH and FeOOH domains and instead points to a cooperative reorganization within a common mixed Ni_*x*_Fe_1–*x*_OOH lattice. Importantly, Ni and Fe K-edge XAS spectra were acquired sequentially from the same electrode spot under identical electrochemical conditions, so the concurrent bond-length contraction at both edges unambiguously reflects a real local restructuring rather than sample heterogeneity. At 1.6 V, the Fe K-edge EXAFS fits yield markedly shortened Fe–O and Fe-M distances, indicating that Fe centers adopt compressed Fe^3+^O_6_ octahedra embedded in the high-valent γ-Ni^3+δ^OOH framework. Together, these results provide direct spectroscopic evidence for the presence of unusually short Fe–O–Ni moieties arising from the intimate structural coupling of Fe and Ni sites under OER conditions.

The above cooperative evolution in TA-CNT-NiFe can be rationalized by considering the local coordination motif compatibility between the Ni and Fe (oxy)­hydroxide motifs formed during activation and their subsequent oxidation-induced distortion under OER potentials. Upon initial reconstruction, isolated Ni^2+^O_6_ and Fe^3+^O_6_ sites evolve into Ni^2+^(OH)_2_ and FeOOH domains composed of edge-sharing MO_6_ octahedra with very similar M-O-M distances (Ni^2+^–O–Ni^2+^ at 3.10 Å; Fe^3+^–O–Fe^3+^ at 3.05–3.12 Å). This close geometric match allows Ni and Fe hydroxides, generated via single-site migration and aggregation on the CNT support, to merge into a solid-solution-like NiFe oxyhydroxide network rather than segregating into discrete phases. As the potential is further increased toward the OER regime, progressive deprotonation of lattice oxygen drives oxidation of Ni^2+^ to high-valent Ni^3+δ^ species and concomitant contraction of the Ni–O and Ni–M distances. Because Fe is already homogeneously distributed within the Ni-centered framework, this oxidation-induced lattice contraction is transmitted to neighboring FeO_6_ octahedra, enforcing a collective distortion of the mixed metal–oxygen network. The result is the formation of compressed FeO_6_ units with shortened Fe–O–Ni linkages that are thermodynamically less favorable than the relaxed FeOOH lattice but become stabilized under high anodic bias due to the large driving force associated with the Ni-centered phase transition (Figure 6). Nevertheless, local strain effects and nanoscale heterogeneity cannot be completely excluded in such dynamically reconstructed oxyhydroxide systems. Because Ni and Fe species are initially dispersed through ligand coordination on the carbon support, the formation of atomic-scale Ni-rich or Fe-rich regions, as well as locally strained interfacial motifs during electrochemical reconstruction, is scientifically plausible. However, we believe that such strained heterojunction-like features are more likely confined to the local atomic scale, while Ni and Fe remain relatively uniformly distributed across the overall nanoscale oxyhydroxide domains. This interpretation is consistent with the highly similar average Fe–O and Ni–O bond distances observed by operando XAS.

**Figure 6:**
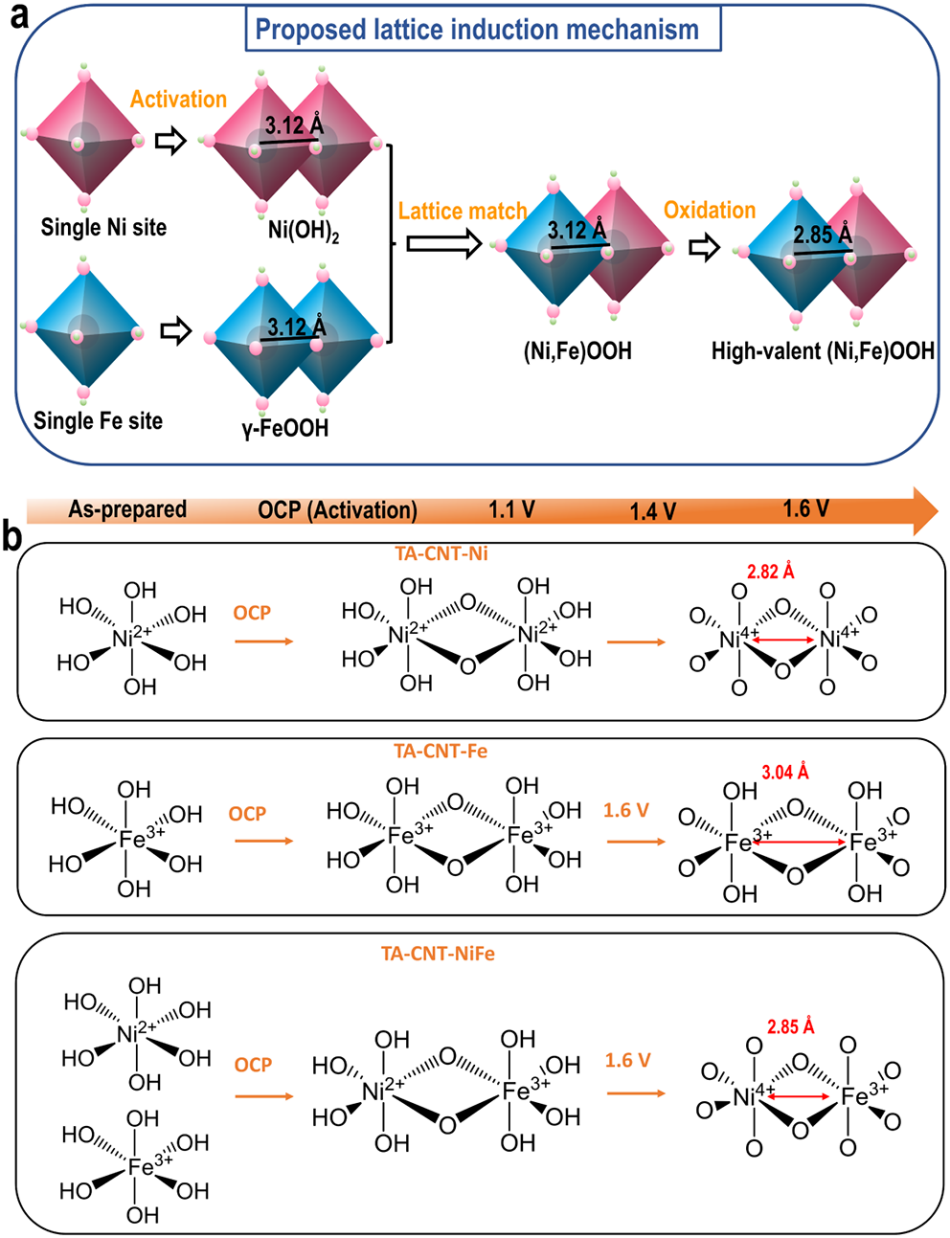
(a) Proposed structural reconstruction pathway and the mechanism of forming short-range mixed high-valent NiFe_1–*x*_OOH active phases. (b) Newly formed active species with different metal–metal distances.

To critically validate this proposed mechanism, we designed a negative-control system in which the two metal oxyhydroxides are structurally incompatible and their oxidation processes are decoupled (Figures S15–17). Co^3+^ was selected as the counter-metal cation because Co-based (oxy)­hydroxides are closely related to the corresponding Ni phases in terms of local coordination geometry and specifically the M-M distances (Table S8). It is worth noting that the CoFe control experiment alone cannot definitively exclude all alternative mechanisms. Rather, the purpose of introducing the CoFe control is to show that the formation of highly compressed Fe–O–M motifs is not a universal feature of all Fe-containing bimetallic OER catalysts but is closely associated with the similarity of short-range local structural parameters during reconstruction. By starting from Co^3+^ precursors, Co is directly transformed into CoOOH without passing through a Ni­(OH)_2_-like Co­(OH)_2_ phase, thereby avoiding the initial formation of a structurally compatible mixed CoFe (oxy)­hydroxide, as well as ensuring that Co oxidation precedes any potential Fe incorporation. *Operando* XANES and EXAFS of the TA-CNT-CoFe catalyst indeed reveal fundamentally different behavior from NiFe: across the entire potential range, the Fe–Fe distance remains essentially constant at ∼2.97 Å, close to that of Fe-only (oxy)­hydroxides (Figure S18, Table S8), while Co–Co distances persist at ∼2.86 Å, with only a minor contribution from a longer Co–Co path around 3.02 Å. No synchronous contraction of Fe-M and Co-M distances is observed, and no shortened Fe–O–Co linkages emerge, indicating that Fe and Co reconstruct into separate FeOOH and CoOOH domains rather than a well-mixed lattice. This behavior is fully consistent with the absence of local M-M distance compatibility and the lack of a cooperative Co-centered phase transition, and therefore provides a stringent falsification test that strongly supports our mechanistic picture derived from the NiFe system (Figure S19). Different reconstruction pathways of Ni- and Co-based systems have been reported experimentally. Operando studies showed that Co-based precatalysts can directly reconstruct into CoOOH-like active phases under OER conditions without a well-defined Co­(OH)_2_ intermediate.^54^ Similarly, our recent studies showed that Co species tend to form CoOOH-like clusters with shorter Co–O–Fe motifs, whereas Ni species preferentially evolve through Ni­(OH)_2_-like intermediates before forming NiOOH phases.^55^ This difference may arise from the lower Co^2+^/Co^3+^ oxidation potential and stronger OH-Co interaction relative to OH-Ni, which facilitate the earlier stabilization of Co–O­(H) environments under anodic conditions.^56^

The formation of such unusually shortened Fe–O–Ni linkages is highly uncommon and stands in clear contrast to most prior operando studies of NiFe (oxy)­hydroxide OER catalysts.^57^ In the majority of reported systems, an increase in Ni oxidation state is accompanied by a pronounced shortening of the Ni^3+δ^ -O bond length, whereas the Fe–O bond length and Fe oxidation state remain largely unchanged throughout the OER potential window.^58,59^ For example, Patzke and coworkers observed contracted Ni–O–Ni motifs in NiFe coordination polymers, but at the Fe edge, only a minor fraction of Fe sites exhibited any indication of shorter Fe–O–Fe/Ni distances.^29^ Similarly, Strasser and coworkers identified high-valent Ni (nominally Ni^4+^) and short Ni–O–Ni distances in NiFe oxides under OER conditions, while the Fe–O coordination remained essentially invariant.^25^ These findings have led to a situation where EXAFS analyses typically resolve distinct Ni–O–M and Fe–O–M distances, and most mechanistic models implicitly assume either a well-mixed Ni_*x*_Fe_1–*x*_OOH phase in short range with well-mixed but electronically inequivalent Ni and Fe sites, or a partially phase-separated arrangement in which only a limited fraction of Ni–O–Fe motifs are formed and Fe largely retains a FeOOH-like environment.^58,60–63^ This contrast suggests that the formation of shortened Fe–O–Ni motifs is not governed by composition alone but is strongly affected by the precursor architecture, ligand/support interactions, and the electrochemical reconstruction pathway. In our system, the atomically dispersed Ni/Fe precursors coordinated by tannic acid on CNTs may provide locally compatible Ni–Fe hydroxide-like motifs before high-valent NiOOH contraction, while the functionalized CNT support helps confine the reconstructed domains and suppress long-range phase segregation. By explicitly demonstrating that (i) true cooperative bond contraction at both metal sites, (ii) the emergence of compressed FeO_6_ octahedra, and (iii) the appearance of shortened Fe–O–Ni linkages occur only when local M-M distance compatibility and high-valent Ni-induced distortion act in concert, and by contrasting this behavior with a structurally mismatched CoFe analogue that does not form such motifs, our study provides a concrete structural framework to rationalize when and how short Fe–O–Ni units can arise. This insight not only clarifies the origin of the rare Fe–O bond shortening reported in a subset of NiFe systems but also underscores that the degree of phase mixing and local lattice compatibility must be explicitly considered when constructing structural and mechanistic models for NiFe and, more broadly, for bimetallic OER catalysts.

### DFT Evaluation of OER Energetics on the Spectroscopically Derived Fe–O–Ni Motif

In the 20 CV cycle tests (Figure 3a), we observed distinct trends in the current density evolution of the TA-CNT-Fe/Ni/NiFe catalysts with increasing cycle numbers. Specifically, the current density of the Fe-based catalyst gradually decreased, whereas the Ni-based catalyst exhibited a slow but steady increase. In contrast, the NiFe catalyst maintained a relatively stable performance (Figure S20). The performance decay of the Fe-based catalyst may arise from the absence of Ni-induced structural stabilization or from its transformation into FeOOH-like phases with intrinsically limited conductivity, as reported previously.^64^ This further suggests that Ni and Fe species in the NiFe catalyst undergo distinct reconstruction under OER conditions, leading to different active-state structures and, consequently, different catalytic activity and stability. The potential influence of Fe impurities from the electrolyte appears negligible, as the OER performance of TA-CNT-Ni in both 1 M commercial and purified KOH remains nearly identical after 1000 CV cycles (Figure S21). The inconspicuous influence of the Fe impurities may be due to the quick phase transition during the first CV cycles, in which the Fe impurities cannot gradually insert into the lattice. Therefore, it is reasonable to infer that the phase transitions play a more critical role in OER performance enhancement. According to the OER performance, the order of the active species in the three catalysts should be Ni–O–Fe > Ni–O–Ni > Fe–O–Fe.

To further support this hypothesis, we conducted DFT calculations for pure FeOOH, NiOOH, Ni­(OH)_2_, defective Ni_*x*_Fe_1–*x*_OOH-D and Ni_*x*_Fe_1–*x*_OOH. The calculated onset potentials for pure FeOOH, NiOOH, Ni­(OH)_2_ Ni_*x*_Fe_1–*x*_OOH-D are η=2.30 V, η=0.95 V, η=1.17 V, and η=1.6 V, respectively (Figures S22–S25). Meanwhile, the calculated onset potentials for TA-CNT-NiFe at the Fe site and the Ni site are η=0.65 V and η=1.0 V, respectively (Figure 7a–b). The results are in very good agreement with the experimental OER performance and strongly suggest that the newly formed Ni–O–Fe species could significantly reduce the activation energy at Fe sites. To further evaluate the catalytic relevance of the spectroscopically suggested NiFeOOH-like structure and clarify how Fe and Ni sites contribute to the OER, we conducted DFT calculations at different metal sites on TA-CNT-NiFe. Three reaction pathways were proposed: (a) Fe site, (b) Ni site, and (c) Ni–Fe dual site. The Ni–Fe dual site enables the two OH^–^ to be absorbed on both Ni and Fe sites parallelly; after the deprotonation on both sites, two adjacent M-O intermediates spontaneously couple to form O_2_. This pathway has been widely reported in NiFe and CoFe dual-site OER catalysts.^65,66^ The details of the calculation are based on the previous method.^14,67^

**Figure 7:**
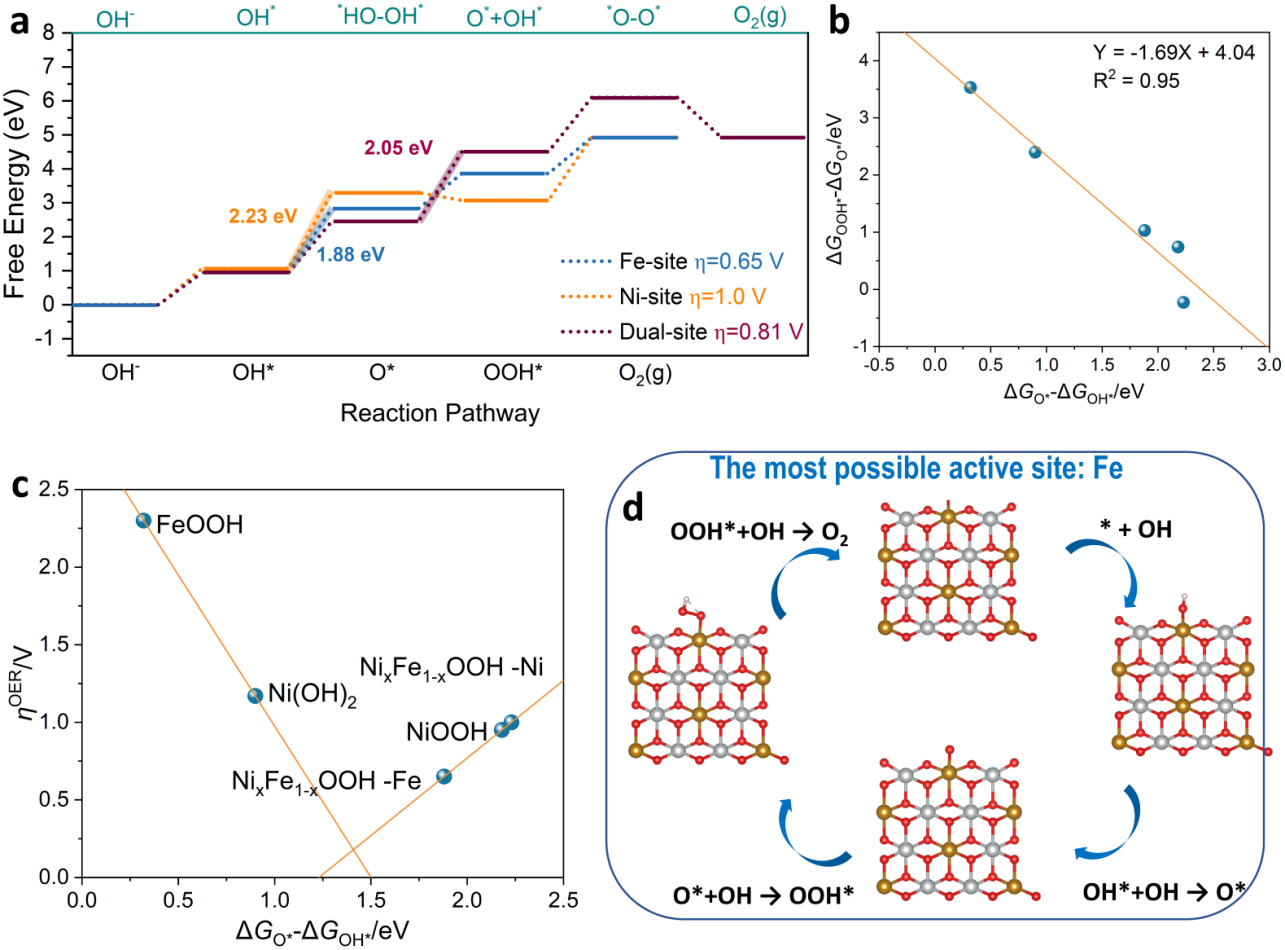
(a) Calculated free energy diagrams of OER intermediates adsorbed on TA-CNT-NiFe; (b) linear relationship between ΔG*OOH-ΔG*O and G*O-ΔG*OH; (c) descriptor of the OER using the *OH→*O step; and (d) the proposed active site.

The calculated potential for TA-CNT-NiFe at the Fe site to drive the OER is η = 0.65 V, which is much lower than that at the Ni site (η=1.0 V) and the dual Ni–Fe site (η=0.81 V). This indicates that the Fe site is more likely to be the active site in TA-CNT-NiFe. It is well-known that *OH, *O, and *OOH species are the key intermediates during the OER. In general, Sabatier’s principle suggests that the adsorption interactions between the active sites and intermediates should not be too strong or too weak. This means that a good OER site should have strong adsorption toward *OH, while other intermediates should have mediate adsorption at the active site, so that the energy difference between each step is not too big. Therefore, only Ni_*x*_Fe_1–*x*_OOH -Fe follows this principle. The energy gaps between all steps are optimized, making the maximum energy only 0.65 V (Table 1). The unusually short Fe–O bond also changes the rate-determining step (RDS) of Fe sites. The RDS for Ni_*x*_Fe_1–*x*_OOH (at both Ni and Fe sites) and NiOOH is *OH to *O, while for FeOOH and Ni­(OH)_2_ it is *O to *OOH (Figures 7a and S22–S25). This indicates that the adsorption strength of *O is crucial for OER. If it is strong, as in FeOOH and Ni­(OH)_2_, ΔG_*O_ will be smaller, resulting in a larger difference in ΔG_*OOH_-ΔG_*O_, which is unfavorable for the reaction. Conversely, if the adsorption is weak, as in Ni_*x*_Fe_1–*x*_OOH (at both Ni and Fe sites) and NiOOH, ΔG_*O_ will be larger, leading to a greater difference in ΔG_*O_-ΔG_*OH_, which is also unfavorable for the reaction. Among these, Ni_*x*_Fe_1–*x*_OOH at the Fe site exhibits moderate *O adsorption strength, making it the most favorable for the OER reaction. Additionally, Figure 7b shows a good linear relationship between ΔG_*OOH_-ΔG_*O_ and G_*O_-ΔG_*OH_, allowing G_*O_-ΔG_*OH_ to predict OER activity in the volcano plot of Figure 7c. Ni_*x*_Fe_1–*x*_OOH at the Fe site is closest to the volcano peak, demonstrating the highest activity. The high activity at the Fe site can be explained by the molecular orbital principle,^68^ which correlates the OER performance with the effective occupation of metal–oxygen antibonding states formed between metal sites and adsorbed *O. In FeOOH, symmetric Fe^3+^–O–Fe^3+^ (HS) coupling results in excessive eg availability at Fe sites, leading to high antibonding state occupation and overly strong *O adsorption (ΔG_*O_ = 1.32 eV, Table 1). In contrast, Ni^3+δ^ OOH, exhibits insufficient eg filling, resulting in weak *O adsorption (ΔG_*O_ = 3.76 eV, Table 1). In Ni_*x*_Fe_1–*x*_OOH, asymmetric Fe^3+^–O–Ni^3+δ^ lattice coupling electronically modulates the effective eg filling of Fe sites through spin exchange interactions,^69,70^ suppressing excessive antibonding occupation while preserving sufficient back-donation. Consequently, *O adsorption at Fe sites becomes Sabatier-optimal (ΔG_*O_ = 2.83 eV, Table 1), minimizing energy barriers between elementary steps and accounting for the superior OER activity (Figure 7d). It is worth noting that the active-site structure shown in Figure 7d should be regarded as a proposed catalytically relevant model rather than a directly identified atomic configuration. This model was constructed based on the local structural parameters obtained from spectroscopic analyses, including the corresponding bond lengths and coordination features of NiOOH-, FeOOH-, and NiFeOOH-like species based on operando spectroscopic evidence. DFT calculations were then used to compare the OER energetics at different metal sites and to assist in the assignment of the most favorable reaction active site. Therefore, the DFT results support that the Fe site in the reconstructed NiFeOOH-like structure is the most catalytically favorable site for OER among the considered models, consistent with the experimental activity trend.

**Table 1:** Free Energies of Different Intermediates Adsorbed on Different Catalysts at Metal Sites^a^

Catalysts	ΔG_*OH_(eV)	ΔG_*O_(eV)	ΔG_*OOH_(eV)	ΔG_*2OH_(eV)	ΔG_*OH*+*O_(eV)	ΔG_*2O_(eV)	η (V)
FeOOH	1.00	1.32	4.85	-	-	-	2.30
NiOOH	1.58	3.76	4.50	-	-	-	0.95
Ni(OH)_2_	0.71	1.61	4.01	-	-	-	1.17
Ni_*x*_Fe_1‑x_OOH-D	0.22	1.63	4.13	-	-	-	1.60
Ni_*x*_Fe_1‑x_OOH -Ni	1.07	3.30	3.07	-	-	-	1.0
Ni_*x*_Fe_1‑x_OOH -Fe	0.95	2.83	3.86	-	-	-	0.65
Dual-site −1	0.95	-	-	2.46	4.50	6.10	0.81
Dual-site −2	0.95	-	-	2.46	3.95	6.10	0.92

a **Note:** Dual-site −1 and −2 mean the first deprotonation at the Ni and Fe sites, respectively.

## Conclusions and Outlooks

In summary, we propose a 6-fold oxygen coordination strategy to successfully synthesize an atomically dispersed NiFe precatalyst as a model system. Atomic-resolution scanning transmission electron microscopy (STEM) and X-ray absorption spectroscopy (XAS) support the atomic dispersion of these active sites. *Operando* XAS studies further reveal that atomic dispersion facilitates the initial formation of Ni­(OH)_2_ and γ-FeOOH during the OER. Due to their structural similarity, these two phases exhibit strong mutual compatibility. As the applied potential increases to 1.6 V vs RHE, γ-FeOOH fully integrates into the high-valent γ-NiOOH phase, yielding a well-mixed Ni_*x*_Fe_1–*x*_OOH structure characterized by an exceptionally short Fe–O bond (∼1.89 Å). Interestingly, comparative studies of monometallic Fe and Ni catalysts, as well as CoFe systems, reveal that Ni induces the formation of a well-mixed Ni–Fe oxyhydroxide phase during surface reconstruction. Based on these observations, we propose a lattice-matching-induced reconstruction mechanism, which provides a fundamental basis for the rational design and synthesis of high-performance NiFe-based OER electrocatalysts. Density functional theory (DFT) calculations further elucidate the electronic origin of the synergistic effect in TA-CNT-NiFe. The electron-deficient Ni^3+δ^ centers introduce a strong localized positive potential, which is transmitted to adjacent Fe^3+^ sites through Ni–O–Fe bridges. This intermetallic charge redistribution reduces the local electron density at Fe, enhances its affinity for OH^–^, and stabilizes key OER intermediates. Consequently, the energy barrier for the rate-determining *O→*OOH step is markedly lowered, significantly boosting the intrinsic OER activity of the Fe sites.

More importantly, our findings suggest a general design principle for multimetallic alkaline OER catalysts: the working active phase should be engineered through the precursor-to-oxyhydroxide reconstruction pathway, rather than by elemental composition alone. In our system, atomically dispersed Ni/Fe precursors, ligand-defined coordination environments, and interfacial confinement by a conductive support promote local Ni–Fe mixing before high-valent NiOOH-like contraction, enabling compressed Fe–O–Ni motifs and enhancing the intrinsic OER activity of Fe sites. This highlights the importance of precursor dispersion, ligand/support interactions, local structural compatibility, and matched anodic reconstruction windows in directing whether different metal species form strongly coupled M-O-M′ motifs or segregated oxyhydroxide domains. Key structural factors such as M-O/M-M bond lengths, ionic size, and coordination geometry should therefore be considered when designing multimetallic precatalysts. This perspective is consistent with previous work showing that different NiFe precatalyst preparation methods can lead to distinct electrochemical conversion pathways and active NiFeOOH structures.^71^ Thus, designing precatalysts with locally compatible coordination motifs and support-confined reconstruction pathways may provide a broadly applicable strategy for constructing highly active and stable multimetallic oxyhydroxide OER catalysts.

## Supplementary Material



## Data Availability

The raw data that support the findings of this study are available from the corresponding authors upon request.

## Notes

Additional experimental section, materials characterizations including XRD, EPR, Raman, STEM-HAADF, and XAS results, additional electrochemical data of operando EIS characterizations, DFT results, and the fitting details of XAS (DOCX)

## ¶

Technical University of Denmark, Department of Physics, Surface Physics and Catalysis, Fysikvej, 2800 Kgs. Lyngby, Denmark. E-mail: wewana@dtu.dk
