# Supplementary material for: Local Coordination Motif Compatibility Induces the Formation of Shortened Fe3+–O–Ni3+δ Moieties as Active Sites for Highly Efficient Oxygen Evolution Reaction
Source: J Am Chem Soc. 2026 Jul 29;148(31):33089–103. doi: 10.1021/jacs.6c05060 (PMC13474943; doi:10.1021/jacs.6c05060)
Supplement: Supplementary file 1 [file ja6c05060_si_001.docx]

**Local Coordination Motif Compatibility Induces the Formation of Shortened Fe^3+^-O-Ni^3+δ^ Moieties as Active Sites for Highly Efficient Oxygen Evolution Reaction**

Wenchao Wan ^1,6*^, Liqun Kang ^1,6*^, Shiqian Wei ^2^, Alexander Schnegg ^1^, Kaltum Abdiaziz ^1^, Longxiang Liu ^3^, Fei Guo ^3^, Christopher S. Allen ^4,5^, Serena DeBeer ^1^, Saskia Heumann ^1*^

1. Max Planck Institute for Chemical Energy Conversion, Stiftstraße 34–36, 45470 Mülheim an der Ruhr, Germany.
2. Leshan West Silicon Materials Photovoltaic New Energy Industry Technology Research Institute, 614000 Leshan, China.
3. Department of Chemistry, University College London, 20 Gordon St, London WC1H 0AJ, United Kingdom.
4. Electron Physical Science Imaging Centre, Diamond Light Source Ltd, Didcot, Oxfordshire, OX11 0DE, United Kingdom.
5. Department of Materials, University of Oxford, Parks Road, Oxford, OX1 3HP, United Kingdom.
6. These authors contributed equally

* Email: [wenchao.wan@cec.mpg.de](mailto:wenchao.wan@cec.mpg.de); [liqun.kang@cec.mpg.de](mailto:liqun.kang@cec.mpg.de); [saskia.heumann@cec.mpg.de](mailto:saskia.heumann@cec.mpg.de)

# Experimental Section

# Materials

Ni(NO_3_)_2_∙6H_2_O (≥99.990 %), Co(NO_3_)_2_∙6H_2_O (≥99.99 %), Fe(NO_3_)_3_∙9H_2_O (≥99.99 %), Tannic acid (C_76_H_52_O_46_) (ACS reagent) were purchased from Sigma-Aldrich. Potassium hydroxide 1 M KOH (1N) volumetric solution was purchased from Fluka. CNT produced by Shandong Dazhan Nano Materials Co. is multi-walled CNT with average diameter around 10 nm. The water used for all experiments is ultra-pure water.

# Synthesis

**Washed CNT:** The multi-walled carbon nanotubes (MWCNTs) used in this study were purchased from Shandong Dazhan Nano Materials Co., with an average diameter of approximately 10 nm. The MWCNTs were first purified via nitric acid washing. Specifically, 20 g of MWCNTs were mixed with 1000 mL of 68% nitric acid, followed by stirring and reflux heating at approximately 100 °C for 20 hours. After the reaction, the gaseous byproducts were purged with nitrogen to remove acidic vapors, facilitating safer handling. The resulting material was then filtered and thoroughly washed overnight in a washing cell with distilled water to eliminate residual acid and impurities such as iron. The cleaned black CNTs were subsequently dried in an oven at 100 °C for 20 hours.

**Tan-CNT-M：**The washed CNTs were mixed with tannic acid and metal precursors-Ni(NO_3_)_2_·6H_2_O and/or Fe(NO_3_)_3_·9H_2_O (for NiFe samples, the molar ratio of Ni to Fe was 2:1)-along with 400 μL of NaOH solution (100 mg/mL) in deionized water. The mixture underwent ultrasonication for 3 hours, followed by continuous stirring overnight. Afterward, the samples were centrifuged to remove the aqueous phase and dried in an oven at 60 °C.

# Electrochemical measurements

Electrochemical water oxidation measurements were performed using a BioLogic VMP3 potentiostat with a standard three-electrode system in 1 M KOH electrolyte. A Hg/HgO electrode filled with 1 M KOH solution and a platinum mesh were used as the reference and counter electrodes, respectively. A carbon cloth (CC) electrode with an area of 1 × 1 cm^2^, loaded with catalyst, served as the working electrode. The CC was sequentially cleaned with 0.1 M HNO_3_, ethanol, and ultra-pure water. A total of 2.5 mg of catalyst was dispersed in 1 mL of ethanol. After 30 minutes of ultrasonication, 50 μL of 5 wt% Nafion solution was added to the dispersion, followed by an additional 60 minutes of sonication to obtain a homogeneous ink. Then, 0.3 mL of the catalyst ink (containing 0.75 mg of catalyst) was drop-cast onto the CC electrode. Prior to polarization curve measurements, all catalyst electrodes were pre-activated by 20 continuous cyclic voltammetry (CV) scans. The scan rates for linear sweep voltammetry (LSV) and CV measurements were set to 10 and 50 mV/s, respectively, over a potential range of 0 to 1.8 V vs. the reversible hydrogen electrode (RHE), in order to minimize capacitive currents. All potentials were referenced to the RHE using the equation: RHE (E_RHE_ = E_Hg/HgO_ + 0.098 + 0.0591×pH V).

# KOH Electrolyte Puriﬁcation

The electrolyte purification was carried out electrochemically using a two-electrode setup, where a NiS_3_–MoS_2_ catalyst was deposited on Ni foam electrodes (3 × 1 cm^2^ geometric surface area, with a high porosity of 95% from Goodfellow). The molar content of the MoS_2_ catalyst on the Ni foam was 8%. During the electrolysis, a constant current of 100 mA was applied in a 1 L standard polystyrene bottle. Over a 12-hour period, samples were collected and analyzed using ICP-OES to measure the residual Fe content. The voltage supplied by the potentiostat was approximately 1.9 V, gradually decreasing throughout the 12-hour electrochemical purification process. This decrease signalled the activation of the Ni foam catalyst and indicated the removal of Fe from the KOH electrolyte, with Fe being incorporated into the Ni foam electrode. After the process, the electrolyte was filtered and stored for further use, while the Ni foam electrodes were thoroughly washed with deionized water and dried under a stream of argon for several minutes.

# Material characterizations

## Powder X-ray diffraction (PXRD)

PXRD patterns were recorded on Bragg-Brentano geometry on a Bruker AXS D8 Advance II theta/theta diffractometer using Cu K_α_ X-ray source.

## Scanning transmission electron microscopy (STEM)

High-resolution aberration-corrected Annular Bright Field (ABF) and High Angle Annular Dark Field (HAADF) STEM images were acquired using a probe-corrected JEOL ARM300CF electron microscope (Instrument E02) of the electron Physical Science Imaging Centre (ePSIC) at Diamond Light Source (DLS, UK). The acceleration voltage for the electron gun was 80 kV. The probe size was set to 8C (Spot 8) with a 30 μm probe-forming aperture (CL aperture) selected, resulting in a probe convergence semi-angle of 24.8 mrad and a beam current of 28.3 pA. The STEM camera length was set to 9.0 cm, which allowed the ADF detector to integrate the scattered electron intensity between 73.7 ± 1.8 and 155.4 ± 1.8 mrad. In addition, a 3 mm aperture was inserted for the BF imaging, corresponding to a semi-angle of 14.8 ± 1.2 mrad (outer angle) for the BF detector. For each sample, a small amount of dry powder was sprinkled on a 400-mesh Cu grid with lacey carbon support film, and the grid was then baked inside a vacuum chamber at 60 ^o^C for 15 minutes. Each sample was exposed to an intense electron beam for 10-15 minutes ('beam shower’) to eliminate the accumulation of carbon contamination during the STEM imaging. Gatan Microscopy Suite software was used for image data acquisition. The FFT image processing and feature extraction/identification were performed using HyperSpy (version 2.3.0),^1^ scikit-image (version 0.25.2)^2^ and SciPy (version 1.15.2)^3^ Python libraries.

## Raman spectroscopy

Ramam spectra were collected on a Thermo Scientific DXR Raman Microscope with a 50 × magnification and 532 nm laser. The measurements before OER were performed using powder samples. Results after OER were acquired using samples dropped on carbon cloth electrodes.

## Electron Paramagnetic Resonance (EPR)

The EPR spectra were obtained using an X-band (~9.64 GHz) Bruker Elexsys E500 EPR spectrometer equipped with a ER4116DM dual mode resonator and an ESR 900 He cryostat. The sample temperature was stabilised at 10 K using a He flow cryostat (Oxford Instruments). The EPR spectra were obtained using a range of microwave power between 20 mW and 6.3 mW for power saturation. The optimized microwave power used for individual samples are mentioned in the corresponding figures. The EPR spectra were recorded at 10 and 30 K with 700 mT field sweep centred at 355 mT, a modulation amplitude of 0.7 mT, a time constant of 40.96 ms, a sweep time of 84 s and a modulation frequency of 100 KHz.

## X-ray Fluorescence (XRF)

XRF results were collected by the Xepos C, Spectro with the measurement time of 10 mins for each sample in the atmosphere (during measurement) of helium/air mixture, using energy range (to be scanned) of 3 keV – 19 keV. Foil (what the bottom of the sample cuvette is made of): Polypropylene 12 µm.

## X-ray absorption spectroscopy (XAS)

The ex situ samples (fresh catalysts) were measured at the SAMBA beamline in SOLEIL (France).^4,5^ At SAMBA beamline, X-rays were generated by a bending magnet, and a Si(220) monochromator operated in QEXAFS mode was used to produce monochromatic beam.^6^ The beam was focused by Pd-coated mirrors and collimated at the sample position to a spot size of approximately 200 μm ×300 μm (V×H) with a photon flux of 3 × 10^12^ ph/s. The energy of the incident beam from DCM was calibrated by assigning the energy of the first inflection in the first derivative XANES of Fe foil, Co foil and Ni foil to 7111.2 eV, 7709 eV and 8333 eV, respectively. The *ex situ* samples were prepared into pellets and measured in transmission mode with the corresponding metal foil measured simultaneously. The intensity of incident beam and the transmitted beam was monitored by ionization chambers (filled with mixture of N_2_ and Ar). For each sample at each absorption edge, three XAS scans were acquired and merged to improve the signal to noise ratio.

The in situ XAS data were collected at the beamline B18 of Diamond light Source (UK).^7,8^ The B18 beamline is equipped with a bending magnet source and a Si(111) DCM operated in QEXAFS mode to produce monochromatic X-ray beam. Through Pt coated mirrors and hamonic rejection mirrors, the X-ray beam was focused and collimated at the sample position with a spot size of 200 μm × 250 μm (V×H). The energy calibration was similar to the SAMBA beamline by measuring the metal foils. A Canberra 36-elements monolithic planar Ge pixel array detector was used for collecting the fluorescence signal in partial fluorescence yield mode (PFY). In situ XAS measurements were performed using in-house designed electrochemical cell. The working electrodes were prepared using the following procedure: 2.5 mg of catalyst was dispersed in 1 mL ethanol. After 30 min of ultrasonication, 50 μL of 5 wt % Nafion solution was further added into the above solution with another 60 min of sonication to form a homogeneous ink. Then 10 μL of the catalyst ink loaded onto a carbon cloth electrode (1cm^2^). All the catalyst electrodes were continuously scanned for 10 CV cycles before measuring polarization curves. The metal loading concentration on the working electrodes were optimized for electrochemical test, which is significantly lower than that of powder/pellet samples for ex situ transmission measurements. Therefore, multiple XAS scan repetitions in fluorescence mode were collected to improve the signal to noise ratio. For each working electrode at each absorption edge, 10-20 scans (80 seconds per scan) were collected from 5-10 different sample spots (spaced horizontally and vertically by 1 mm).

The XAS spectra were analyzed using the Demeter software package (including Athena and Artemis programs, version 0.9.26).^9^ Pre-edge background subtraction and post-edge normalization of the XAFS data were performed using the Athena program. A linear regression background was determined, and a quadratic polynomial regression for post-edge normalization was applied. The spectra were splined from k=0 Å^-1^ to k=12.5 Å^-1^ with rbkg of 1.0 and k-weight of 2. The fitting of EXAFS spectra (R range: 1 to 3 Å, k range: 3.0 to 11.5 Å^-1^) was performed using the Artemis program based on scattering paths generated from FEFF6. The amplitude reduction factors S_0_^2^ were determined by fitting of k^2^-weighted R-space EXAFS of Fe foil, Co foil and Ni foil for each XAS edge respectively. The S_0_^2^ values for Fe K-edge (0.75), Co K-edge (0.72) and Ni K-edge (0.82) were used as fixed parameter for the EXAFS fitting of other samples. The wavelet transform EXAFS spectra were produced using the Cauchy wavelet transform function built in the xraylarch python library (version 0.9.74).^10,11^

The XAFS of α-FeOOH, γ-FeOOH, Fe(OH)_3_, CoOOH, Co(OH)_2_, NiOOH and Ni(OH)_2_ were standard spectra indexed in the BL14B2 XAFS Standard Sample Database,^12^ and the energy calibration was performed by aligning the XANES of Fe/Co/Ni foils measured at BL14B2, SAMBA and B18 beamline.

**DFT calculations**

All spin-polarization DFT calculations were performed in the Vienna ab initio simulation package (VASP) with the projector-augmented wave (PAW) to describe the core-electron interactions.^13^ The exchange-correlation energies were calculated by the Perdew, Burke and Ernzerhof (PBE) method based on the generalized gradient approximation (GGA).^14^ Given the limitations of GGA/PBE methods to describe the van der Waals forces, the dispersion force corrected DFT (DFT-D) method introduced by Grimme was employed.^15^ The cut-off energy of 450 eV and the k-point mesh of 2×2×1 were used for all calculations in this work. The convergence thresholds of energies and forces were1×10^-4^ eV and 0.05 eV/Å, respectively. A vacuum space of 15 Å was inserted to separate the interaction between the periodic calculation structures. Structure with oxygen defects was created by removing one oxygen atom between Ni and Fe atom. The creation of the structures for Fe, Ni and NiFe hydroxides and the OER pathways were according to the previous work of Nørskov et al.^16^

# Supplementary Figures

**Figure S1.** (a) XRD patterns of the as-prepared catalysts, tannic acid and CNT. (b) Raman spectra of the as-prepared catalysts.

**Figure S2.** Wavelet transforms of the k^2^-weighted EXAFS signals : (a) TA-CNT-Ni at Ni K-edge, (b) TA-CNT-Fe at Fe K-edge, (c) TA-CNT-NiFe at Ni K-edge and (d) TA-CNT-NiFe at Fe K-edge.

**Figure S3.** cw X-band EPR spectra of TA-CNT-M series. Spectra are plotted offset for better visibility. Measurement parameters: *T* = 10 K, MW frequency: 9.64 GHz, MW power: 2 mW, modulation amplitude: 0.7 mT.

**Figure S4.** OER performance of the catalysts normalized by mass.

**Figure S5.** *Operando* EIS characterizations of TA-CNT-Fe, TA-CNT-Ni and TA-CNT-NiFe for the OER; (a, c, e) Nyquist plots and (b, d, f) Bode plots.

**Figure S6.** Photo of the electrochemical cell designed for the In situ XAS measurements.

**Figure S7.** (a) In situ XANES spectra of TA-CNT-Ni collected at different potentials and the reference compounds. (b) Plot of the shift of the absorption energies of XANES at different potentials, and comparison with the similar reference compounds. (c) K pre-edge spectra of the TA-CNT-Ni at different potentials and comparison with the similar reference compounds.

**Figure S8.** (a) In situ XANES spectra of TA-CNT-Fe collected at different potentials and the reference compounds. (b) K pre-edge spectra of the TA-CNT-Fe at different potentials and comparison with the similar reference compounds.

**Figure S9.** Experimental Ni K-edge Fourier transform EXAFS signals of TA-CNT-Ni.

**Figure S10.** Experimental Fe K-edge Fourier transform EXAFS signals of TA-CNT-Fe.

**Figure S11**. Wavelet transforms for the *k^2^*-weighted EXAFS signals of TA-CNT-NiFe at 1.6 V vs. RHE. (a) Ni K-edge, (b) Fe K-edge.

**Figure S12.** Raman spectra of TA-CNT-NiFe recorded right after OER.

**Figure S13.** Raman spectra of TA-CNT-Ni recorded right after OER.

**Figure S14.** Raman spectra of TA-CNT-Fe recorded right after OER.

**Figure S15.** (a) In situ Fe K edge XANES spectra of TA-CNT-CoFe collected at different potentials and the reference compounds. (b) Pre-edge spectra of the TA-CNT-CoFe at different potentials and comparison with the similar reference compounds. (c) Experimental Fe K-edge Fourier transform EXAFS signals of TA-CNT-CoFe.

**Figure S16.** (a) In situ Co K edge XANES spectra of TA-CNT-CoFe collected at different potentials and the reference compounds. (b) K pre-edge spectra at different potentials and comparison with the similar reference compounds. (c) Plot of the shift of the absorption energies of XANES at different potentials, and comparison with the similar reference compounds.

**Figure S17.** Experimental Co K-edge Fourier transform EXAFS signals of TA-CNT-CoFe.

**Figure S****18.** HAADF-STEM images of TA-CNT-CoFe after in situ XAS measurements.

**Figure S19.** The structural reconstruction and proposed phases transition mechanism of TA-CNT-CoFe.

**Figure S20.** Real time monitor of the current density during the CV tests of TA-CNT-Ni, TA-CNT-Fe, TA-CNT-NiFe.

**Figure S21.** 1000 CV cycles test of TA-CNT-Ni 1M commercial and purified KOH.

**Figure S22.** Calculated free energy diagrams of OER intermediates adsorbed on TA-CNT-Fe.

**Figure S23.** Calculated free energy diagrams of OER intermediates adsorbed on TA-CNT-Ni.

**Figure S24.** Calculated free energy diagrams of OER intermediates adsorbed on Ni(OH)_2_.

**Figure S25.** Calculated free energy diagrams of OER intermediates adsorbed on Ni_x_Fe_1-x_OOH-D.

**All the fitting details of XAS data**

**Figure S26.** Fitting results of Fe K-edge k^2^-weighted k-space and R-space FT-EXAFS spectra of Fe foil in (a) k-space, (b) R-space magnitude, (c) R-space real part and (d) R-space imaginary part. The R-space spectra are plotted without phase correction.

**Figure S27.** Fitting results of Fe K-edge k^2^-weighted k-space and R-space FT-EXAFS spectra of TA-CNT-Fe in (a) k-space, (b) R-space magnitude, (c) R-space real part and (d) R-space imaginary part. The R-space spectra are plotted without phase correction.

**Figure S28.** Fitting results of Fe K-edge k^2^-weighted k-space and R-space FT-EXAFS spectra of TA-CNT-CoFe in (a) k-space, (b) R-space magnitude, (c) R-space real part and (d) R-space imaginary part. The R-space spectra are plotted without phase correction.

**Figure S29.** Fitting results of Fe K-edge k^2^-weighted k-space and R-space FT-EXAFS spectra of TA-CNT-NiFe in (a) k-space, (b) R-space magnitude, (c) R-space real part and (d) R-space imaginary part. The R-space spectra are plotted without phase correction.

**Figure S30.** Fitting results of Co K-edge k^2^-weighted k-space and R-space FT-EXAFS spectra of Co foil in (a) k-space, (b) R-space magnitude, (c) R-space real part and (d) R-space imaginary part. The R-space spectra are plotted without phase correction.

**Figure S31.** Fitting results of Co K-edge k^2^-weighted k-space and R-space FT-EXAFS spectra of TA-CNT-Co in (a) k-space, (b) R-space magnitude, (c) R-space real part and (d) R-space imaginary part. The R-space spectra are plotted without phase correction.

**Figure S32.** Fitting results of Co K-edge k^2^-weighted k-space and R-space FT-EXAFS spectra of TA-CNT-CoFe in (a) k-space, (b) R-space magnitude, (c) R-space real part and (d) R-space imaginary part. The R-space spectra are plotted without phase correction.

**Figure S33.** Fitting results of Ni K-edge k^2^-weighted k-space and R-space FT-EXAFS spectra of Ni foil in (a) k-space, (b) R-space magnitude, (c) R-space real part and (d) R-space imaginary part. The R-space spectra are plotted without phase correction.

**Figure S34.** Fitting results of Ni K-edge k^2^-weighted k-space and R-space FT-EXAFS spectra of TA-CNT-Ni in (a) k-space, (b) R-space magnitude, (c) R-space real part and (d) R-space imaginary part. The R-space spectra are plotted without phase correction.

**Figure S35.** Fitting results of Ni K-edge k^2^-weighted k-space and R-space FT-EXAFS spectra of TA-CNT-NiFe in (a) k-space, (b) R-space magnitude, (c) R-space real part and (d) R-space imaginary part. The R-space spectra are plotted without phase correction.

**Figure S36.** Fitting results of Fe K-edge k^2^-weighted k-space and R-space FT-EXAFS spectra of TA-CNT-Fe under OCP condition in (a) k-space, (b) R-space magnitude, (c) R-space real part and (d) R-space imaginary part. The R-space spectra are plotted without phase correction.

**Figure S37.** Fitting results of Fe K-edge k^2^-weighted k-space and R-space FT-EXAFS spectra of TA-CNT-Fe at 1.1 V vs RHE in (a) k-space, (b) R-space magnitude, (c) R-space real part and (d) R-space imaginary part. The R-space spectra are plotted without phase correction.

**Figure S38.** Fitting results of Fe K-edge k^2^-weighted k-space and R-space FT-EXAFS spectra of TA-CNT-Fe at 1.4 V vs RHE in (a) k-space, (b) R-space magnitude, (c) R-space real part and (d) R-space imaginary part. The R-space spectra are plotted without phase correction.

**Figure S39.** Fitting results of Fe K-edge k^2^-weighted k-space and R-space FT-EXAFS spectra of TA-CNT-Fe at 1.6 V vs RHE in (a) k-space, (b) R-space magnitude, (c) R-space real part and (d) R-space imaginary part. The R-space spectra are plotted without phase correction.

**Figure S40.** Fitting results of Fe K-edge k^2^-weighted k-space and R-space FT-EXAFS spectra of TA-CNT-CoFe under OCP condition in (a) k-space, (b) R-space magnitude, (c) R-space real part and (d) R-space imaginary part. The R-space spectra are plotted without phase correction.

**Figure S41.** Fitting results of Fe K-edge k^2^-weighted k-space and R-space FT-EXAFS spectra of TA-CNT-CoFe at 1.1 V vs RHE in (a) k-space, (b) R-space magnitude, (c) R-space real part and (d) R-space imaginary part. The R-space spectra are plotted without phase correction.

**Figure S42.** Fitting results of Fe K-edge k^2^-weighted k-space and R-space FT-EXAFS spectra of TA-CNT-CoFe at 1.4 V vs RHE in (a) k-space, (b) R-space magnitude, (c) R-space real part and (d) R-space imaginary part. The R-space spectra are plotted without phase correction.

**Figure S43.** Fitting results of Fe K-edge k^2^-weighted k-space and R-space FT-EXAFS spectra of TA-CNT-CoFe at 1.6 V vs RHE in (a) k-space, (b) R-space magnitude, (c) R-space real part and (d) R-space imaginary part. The R-space spectra are plotted without phase correction.

**Figure S44.** Fitting results of Fe K-edge k^2^-weighted k-space and R-space FT-EXAFS spectra of TA-CNT-NiFe under OCP condition in (a) k-space, (b) R-space magnitude, (c) R-space real part and (d) R-space imaginary part. The R-space spectra are plotted without phase correction.

**Figure S45.** Fitting results of Fe K-edge k^2^-weighted k-space and R-space FT-EXAFS spectra of TA-CNT-NiFe at 1.1 V vs RHE in (a) k-space, (b) R-space magnitude, (c) R-space real part and (d) R-space imaginary part. The R-space spectra are plotted without phase correction.

**Figure S46.** Fitting results of Fe K-edge k^2^-weighted k-space and R-space FT-EXAFS spectra of TA-CNT-NiFe at 1.4 V vs RHE in (a) k-space, (b) R-space magnitude, (c) R-space real part and (d) R-space imaginary part. The R-space spectra are plotted without phase correction.

**Figure S47.** Fitting results of Fe K-edge k^2^-weighted k-space and R-space FT-EXAFS spectra of TA-CNT-NiFe at 1.6 V vs RHE in (a) k-space, (b) R-space magnitude, (c) R-space real part and (d) R-space imaginary part. The R-space spectra are plotted without phase correction.

**Figure S48.** Fitting results of Co K-edge k^2^-weighted k-space and R-space FT-EXAFS spectra of TA-CNT-CoFe under OCP condition in (a) k-space, (b) R-space magnitude, (c) R-space real part and (d) R-space imaginary part. The R-space spectra are plotted without phase correction.

**Figure S49.** Fitting results of Co K-edge k^2^-weighted k-space and R-space FT-EXAFS spectra of TA-CNT-CoFe at 1.1 V vs RHE in (a) k-space, (b) R-space magnitude, (c) R-space real part and (d) R-space imaginary part. The R-space spectra are plotted without phase correction.

**Figure S50.** Fitting results of Co K-edge k^2^-weighted k-space and R-space FT-EXAFS spectra of TA-CNT-CoFe at 1.4 V vs RHE in (a) k-space, (b) R-space magnitude, (c) R-space real part and (d) R-space imaginary part. The R-space spectra are plotted without phase correction.

**Figure S51.** Fitting results of Co K-edge k^2^-weighted k-space and R-space FT-EXAFS spectra of TA-CNT-CoFe at 1.6 V vs RHE in (a) k-space, (b) R-space magnitude, (c) R-space real part and (d) R-space imaginary part. The R-space spectra are plotted without phase correction.

**Figure S52.** Fitting results of Ni K-edge k^2^-weighted k-space and R-space FT-EXAFS spectra of TA-CNT-Ni at under OCP condition in (a) k-space, (b) R-space magnitude, (c) R-space real part and (d) R-space imaginary part. The R-space spectra are plotted without phase correction.

**Figure S53.** Fitting results of Ni K-edge k^2^-weighted k-space and R-space FT-EXAFS spectra of TA-CNT-Ni at 1.1 V vs RHE in (a) k-space, (b) R-space magnitude, (c) R-space real part and (d) R-space imaginary part. The R-space spectra are plotted without phase correction.

**Figure S54.** Fitting results of Ni K-edge k^2^-weighted k-space and R-space FT-EXAFS spectra of TA-CNT-Ni at 1.4 V vs RHE in (a) k-space, (b) R-space magnitude, (c) R-space real part and (d) R-space imaginary part. The R-space spectra are plotted without phase correction.

**Figure S55.** Fitting results of Ni K-edge k^2^-weighted k-space and R-space FT-EXAFS spectra of TA-CNT-Ni at 1.6 V vs RHE in (a) k-space, (b) R-space magnitude, (c) R-space real part and (d) R-space imaginary part. The R-space spectra are plotted without phase correction.

**Figure S56.** Fitting results of Ni K-edge k^2^-weighted k-space and R-space FT-EXAFS spectra of TA-CNT-NiFe under OCP condition in (a) k-space, (b) R-space magnitude, (c) R-space real part and (d) R-space imaginary part. The R-space spectra are plotted without phase correction.

**Figure S57.** Fitting results of Ni K-edge k^2^-weighted k-space and R-space FT-EXAFS spectra of TA-CNT-NiFe at 1.1 V vs RHE in (a) k-space, (b) R-space magnitude, (c) R-space real part and (d) R-space imaginary part. The R-space spectra are plotted without phase correction.

**Figure S58.** Fitting results of Ni K-edge k^2^-weighted k-space and R-space FT-EXAFS spectra of TA-CNT-NiFe at 1.4 V vs RHE in (a) k-space, (b) R-space magnitude, (c) R-space real part and (d) R-space imaginary part. The R-space spectra are plotted without phase correction.

**Figure S59.** Fitting results of Ni K-edge k^2^-weighted k-space and R-space FT-EXAFS spectra of TA-CNT-NiFe at 1.6 V vs RHE in (a) k-space, (b) R-space magnitude, (c) R-space real part and (d) R-space imaginary part. The R-space spectra are plotted without phase correction.

**Figure S60.** Position 1: The interplanar d-spacings of TA-CNT-Ni identified after in situ XAS measurements, together with the assignment of potential crystallographic facets based on standard diffraction references.

**Figure S61**. Position 2: The interplanar d-spacings of TA-CNT-Ni identified after in situ XAS measurements, together with the assignment of potential crystallographic facets based on standard diffraction references.

**Figure S62**. Position 3: The interplanar d-spacings of TA-CNT-Ni identified after in situ XAS measurements, together with the assignment of potential crystallographic facets based on standard diffraction references.

**Figure S63**. Position 4: The interplanar d-spacings of TA-CNT-Ni identified after in situ XAS measurements, together with the assignment of potential crystallographic facets based on standard diffraction references.

**Figure S64**. Position 1: The interplanar d-spacings of TA-CNT-Fe identified after in situ XAS measurements, together with the assignment of potential crystallographic facets based on standard diffraction references.

**Figure S65**. Position 2: The interplanar d-spacings of TA-CNT-Fe identified after in situ XAS measurements, together with the assignment of potential crystallographic facets based on standard diffraction references.

**Figure S66**. Position 3: The interplanar d-spacings of TA-CNT-Fe identified after in situ XAS measurements, together with the assignment of potential crystallographic facets based on standard diffraction references.

**Figure S67**. Position 4: The interplanar d-spacings of TA-CNT-Fe identified after in situ XAS measurements, together with the assignment of potential crystallographic facets based on standard diffraction references.

**Figure S68**. Position 5: The interplanar d-spacings of TA-CNT-Fe identified after in situ XAS measurements, together with the assignment of potential crystallographic facets based on standard diffraction references.

**Figure S69**. Position 6: The interplanar d-spacings of TA-CNT-Fe identified after in situ XAS measurements, together with the assignment of potential crystallographic facets based on standard diffraction references.

**Figure S70**. Position 7: The interplanar d-spacings of TA-CNT-Fe identified after in situ XAS measurements, together with the assignment of potential crystallographic facets based on standard diffraction references.

**Figure S71**. Position 8: The interplanar d-spacings of TA-CNT-Fe identified after in situ XAS measurements, together with the assignment of potential crystallographic facets based on standard diffraction references.

**Figure S72**. Position 1: The interplanar d-spacings of TA-CNT-NiFe identified after in situ XAS measurements, together with the assignment of potential crystallographic facets based on standard diffraction references.

**Figure S73**. Position 2: The interplanar d-spacings of TA-CNT-NiFe identified after in situ XAS measurements, together with the assignment of potential crystallographic facets based on standard diffraction references.

**Figure S74**. Position 3: The interplanar d-spacings of TA-CNT-NiFe identified after in situ XAS measurements, together with the assignment of potential crystallographic facets based on standard diffraction references.

**Figure S75**. Position 4: The interplanar d-spacings of TA-CNT-NiFe identified after in situ XAS measurements, together with the assignment of potential crystallographic facets based on standard diffraction references.

**Figure S76**. Position 5: The interplanar d-spacings of TA-CNT-NiFe identified after in situ XAS measurements, together with the assignment of potential crystallographic facets based on standard diffraction references.

**Figure S77**. Position 6: The interplanar d-spacings of TA-CNT-NiFe identified after in situ XAS measurements, together with the assignment of potential crystallographic facets based on standard diffraction references.

**Figure S78**. Position 7: The interplanar d-spacings of TA-CNT-NiFe identified after in situ XAS measurements, together with the assignment of potential crystallographic facets based on standard diffraction references.

**Figure S79**. Position 8: The interplanar d-spacings of TA-CNT-NiFe identified after in situ XAS measurements, together with the assignment of potential crystallographic facets based on standard diffraction references.

**Figure S80**. Position 9: The interplanar d-spacings of TA-CNT-NiFe identified after in situ XAS measurements, together with the assignment of potential crystallographic facets based on standard diffraction references.

**Figure S81**. Position 10: The interplanar d-spacings of TA-CNT-NiFe identified after in situ XAS measurements, together with the assignment of potential crystallographic facets based on standard diffraction references.

**Figure S82**. Position 11: The interplanar d-spacings of TA-CNT-NiFe identified after in situ XAS measurements, together with the assignment of potential crystallographic facets based on standard diffraction references.

**Figure 83.** HAADF-STEM images of TA-CNT-Fe and TA-CNT-NiFe after operando XAS measurements.

**Standard γ-NiOOH diffraction crystallographic facets**

**Standard β-NiOOH diffraction crystallographic facets**

**Standard β-N(OH)_2_ diffraction crystallographic facets**

**Standard β-FeOOH diffraction crystallographic facets**

**Standard γ -FeOOH diffraction crystallographic facets**

# Supplementary Tables

**Table S1.** The metal contents of TA-CNT-Fe, TA-CNT-Ni, TA-CNT-Co, TA-CNT-CoFe and TA-CNT-NiFe acquired by X-ray fluorescence (XRF) measurements.

| **Samples** | **Fe (wt. %)** | **Co (wt.%)** | **Ni (wt.%)** |
| --- | --- | --- | --- |
| **TA-CNT-Fe** | 0.83 |  |  |
| **TA-CNT-Ni** |  |  | 1.5 |
| **TA-CNT-Co** |  | 1.97 |  |
| **TA-CNT-NiFe** | 0.46 |  | 0.81 |
| **TA-CNT-CoFe** | 0.31 | 0.59 |  |

**Table S2.** EXAFS Fitting parameters of all ex situ samples. Main interatomic distances, atomic coordination numbers (CN) and Debye-Waller factors (σ^2^) calculated from Artemis fitting of the experimental FT|*k*^2^χ(*k*)| spectra. *S*_0_^2^ denotes the amplitude reduction factor and Δ*E*_0_ the energy shift.

| **Absorption**  **Edge** | **Sample** | **Scattering**  **path** | **C.N.** | **R [Å]** | **σ^2^ [Å^2^]** | **E_0_ [eV]** | **R-factor** |
| --- | --- | --- | --- | --- | --- | --- | --- |
| Fe K-edge | Fe foil | Fe-Fe | 8 (fixed) | 2.46 ± 0.01 | 0.0051 ± 0.0007 | 7117.1 ± 1.2 | 0.0067 |
|  |  | Fe-Fe | 6 (fixed) | 2.84 ± 0.01 |  |  |  |
|  | TA-CNT-Fe | Fe-O | 6.0 ± 0.4 | 2.00 ± 0.01 | 0.0066 ± 0.0010 | 7125.6 ± 0.9 | 0.0026 |
|  |  | Fe-C | 2.2 ± 0.6 | 2.80 ± 0.03 |  |  |  |
|  | TA-CNT-CoFe | Fe-O | 6.4 ± 0.7 | 2.00 ± 0.01 | 0.0079 ± 0.0016 | 7125.4 ± 1.8 | 0.0055 |
|  |  | Fe-C | 2.3 ± 1.0 | 2.70 ± 0.04 |  |  |  |
|  | TA-CNT-NiFe | Fe-O | 6.3 ± 0.9 | 2.01 ± 0.01 | 0.0071 ± 0.0019 | 7126.6 ± 2.0 | 0.0097 |
|  |  | Fe-C | 1.7 ± 1.3 | 2.76 ± 0.06 |  |  |  |
| Co K-edge | Co foil | Co-Co | 12 (fixed) | 2.49 ± 0.01 | 0.0059 ± 0.0003 | 7716.1 ± 0.4 | 0.0023 |
|  | TA-CNT-Co | Co-O | 6.5 ± 0.4 | 2.07 ± 0.01 | 0.0072 ± 0.0009 | 7718.6 ± 0.7 | 0.0021 |
|  |  | Co-C | 2.0 ± 0.5 | 2.91 ± 0.02 |  |  |  |
|  | TA-CNT-CoFe | Co-O | 6.6 ± 0.5 | 2.07 ± 0.01 | 0.0081 ± 0.0011 | 7718.5 ± 1.0 | 0.0030 |
|  |  | Co-C | 1.0 ± 0.7 | 2.90 ± 0.03 |  |  |  |
| Ni K-edge | Ni foil | Ni-Ni | 12 (fixed) | 2.48 ± 0.01 | 0.0061 ± 0.0003 | 8339.5 ± 0.4 | 0.0019 |
|  | TA-CNT-Ni | Ni-O | 6.2 ± 0.4 | 2.04 ± 0.01 | 0.0064 ± 0.0008 | 8341.9 ± 0.8 | 0.0021 |
|  |  | Ni-C | 1.9 ± 0.6 | 2.84 ± 0.03 |  |  |  |
|  | TA-CNT-NiFe | Ni-O | 6.2 ± 0.2 | 2.04 ± 0.01 | 0.0055 ± 0.0007 | 8341.9 ± 0.9 | 0.0042 |
|  |  | Ni-C | 1.2 ± 0.7 | 2.85 ± 0.05 |  |  |  |

**Table S3.** EXAFS fitting parameters of all *in situ* samplesd. Main interatomic distances, atomic coordination numbers (CN) and Debye-Waller factors (σ^2^) calculated from Artemis fitting of the experimental FT|*k*^2^χ(*k*)| spectra. *S*_0_^2^ denotes the amplitude reduction factor and Δ*E*_0_ the energy shift.

| **Absorption**  **Edge** | **Sample** | **Scattering**  **path** | **C.N.** | **R [Å]** | **σ^2^ [Å^2^]** | **E_0_ [eV]** | **R-factor** |
| --- | --- | --- | --- | --- | --- | --- | --- |
| Fe K-edge | TA-CNT-Fe-OCP | Fe-O | 5.7 ± 0.5 | 1.96 ± 0.01 | 0.0093 ± 0.0014 | 7123.2 ± 1.0 | 0.0073 |
|  |  | Fe-Fe | 5.2 ± 0.7 | 3.04 ± 0.02 | 0.0133 ± 0.0021 |  |  |
|  | TA-CNT-Fe-1.1V | Fe-O | 6.3 ± 0.6 | 1.97 ± 0.01 | 0.0106 ± 0.0015 | 7123.9 ± 1.0 | 0.0055 |
|  |  | Fe-Fe | 5.8 ± 0.8 | 3.05 ± 0.01 | 0.0147 ± 0.0023 |  |  |
|  | TA-CNT-Fe-1.4V | Fe-O | 6.1 ± 0.5 | 1.97 ± 0.01 | 0.0103 ± 0.0015 | 7124.3 ± 0.9 | 0.0054 |
|  |  | Fe-Fe | 5.7 ± 0.8 | 3.05 ± 0.01 | 0.0140 ± 0.0020 |  |  |
|  | TA-CNT-Fe-1.6V | Fe-O | 5.8 ± 0.3 | 1.96 ± 0.01 | 0.0091 ± 0.0010 | 7123.4 ± 0.7 | 0.0045 |
|  |  | Fe-Fe | 6.0 ± 0.5 | 3.04 ± 0.02 | 0.0129 ± 0.0017 |  |  |
|  | TA-CNT-CoFe-OCP | Fe-O | 6.1 ± 0.8 | 1.97 ± 0.01 | 0.0103 ± 0.0017 | 7123.8 ± 0.9 | 0.0046 |
|  |  | Fe-Fe/Co | 4.7 ± 0.8 | 2.99 ± 0.01 | 0.0134 ± 0.0023 |  |  |
|  | TA-CNT-CoFe-1.1V | Fe-O | 5.8 ± 0.7 | 1.96 ± 0.01 | 0.0101 ± 0.0017 | 7122.1 ± 1.0 | 0.0094 |
|  |  | Fe-Fe/Co | 4.6 ± 0.7 | 2.96 ± 0.01 | 0.0140 ± 0.0025 |  |  |
|  | TA-CNT-CoFe-1.4V | Fe-O | 6.1 ± 1.0 | 1.98 ± 0.01 | 0.0114 ± 0.0024 | 7124.8 ± 1.1 | 0.0094 |
|  |  | Fe-Fe/Co | 4.5 ± 1.0 | 2.98 ± 0.01 | 0.0136 ± 0.0030 |  |  |
|  | TA-CNT-CoFe-1.6V | Fe-O | 5.7 ± 0.8 | 1.94 ± 0.01 | 0.0105 ± 0.0021 | 7121.0 ± 1.4 | 0.0122 |
|  |  | Fe-Fe/Co | 4.4 ± 0.9 | 2.94 ± 0.02 | 0.0147 ± 0.0034 |  |  |
|  | TA-CNT-NiFe-OCP | Fe-O | 6.1 ± 0.4 | 2.00 ± 0.01 | 0.0052 ± 0.0009 | 7124.3 ± 0.8 | 0.0037 |
|  |  | Fe-Fe/Ni | 4.1 ± 0.5 | 3.12 ± 0.01 |  |  |  |
|  | TA-CNT-NiFe-1.1V | Fe-O | 5.9 ± 0.9 | 1.99 ± 0.01 | 0.0050 ± 0.0010 | 7124.2 ± 0.9 | 0.0067 |
|  |  | Fe-Fe/Ni | 4.1 ± 0.6 | 3.12 ± 0.01 |  |  |  |
|  | TA-CNT-NiFe-1.4V | Fe-O | 6.1 ± 0.6 | 2.00 ± 0.01 | 0.0053 ± 0.0012 | 7124.4 ± 1.2 | 0.0071 |
|  |  | Fe-Fe/Ni | 4.0 ± 0.6 | 3.12 ± 0.02 |  |  |  |
|  | TA-CNT-NiFe-1.6V | Fe-O | 5.7 ± 0.4 | 1.91 ± 0.01 | 0.0085 ± 0.0010 | 7121.9 ± 1.4 | 0.0047 |
|  |  | Fe-Fe/Ni | 5.1 ± 0.8 | 2.86 ± 0.01 | 0.0055 ± 0.0007 |  |  |
|  |  | Fe-Fe/Ni | 2.3 ± 0.6 | 3.08 ± 0.02 |  |  |  |
| Co K-edge | TA-CNT-CoFe-OCP | Co-O | 5.7 ± 0.4 | 1.92 ± 0.01 | 0.0027 ± 0.0010 | 7723.2 ± 1.0 | 0.0125 |
|  |  | Co-Fe/Co | 4.6 ± 0.7 | 2.85 ± 0.01 |  |  |  |
|  |  | Co-Fe/Co | 1.7 ± 0.4 | 3.02 ± 0.02 |  |  |  |
|  | TA-CNT-CoFe-1.1V | Co-O | 5.7 ± 0.3 | 1.92 ± 0.01 | 0.0029 ± 0.0007 | 7723.5 ± 0.7 | 0.006 |
|  |  | Co-Fe/Co | 4.8 ± 0.5 | 2.85 ± 0.01 |  |  |  |
|  |  | Co-Fe/Co | 1.9 ± 0.3 | 3.02 ± 0.02 |  |  |  |
|  | TA-CNT-CoFe-1.4V | Co-O | 5.7 ± 0.4 | 1.91 ± 0.01 | 0.0028 ± 0.0008 | 7723.3 ± 0.8 | 0.0085 |
|  |  | Co-Fe/Co | 4.8 ± 0.6 | 2.84 ± 0.01 |  |  |  |
|  |  | Co-Fe/Co | 1.5 ± 0.4 | 3.00 ± 0.02 |  |  |  |
|  | TA-CNT-CoFe-1.6V | Co-O | 6.1 ± 0.5 | 1.91 ± 0.01 | 0.0035 ± 0.0010 | 7723.1 ± 1.0 | 0.0089 |
|  |  | Co-Fe/Co | 5.0 ± 0.8 | 2.85 ± 0.01 |  |  |  |
|  |  | Co-Fe/Co | 1.2 ± 0.5 | 3.02 ± 0.04 |  |  |  |
| Ni K-edge | TA-CNT-Ni-OCP | Ni-O | 5.7 ± 0.3 | 2.05 ± 0.01 | 0.0068 ± 0.0008 | 8341.3 ± 0.6 | 0.0063 |
|  |  | Ni-Ni | 5.2 ± 0.5 | 3.10 ± 0.01 |  |  |  |
|  | TA-CNT-Ni-1.1V | Ni-O | 5.5 ± 0.2 | 2.06 ± 0.01 | 0.0062 ± 0.0007 | 8341.5 ± 0.6 | 0.0066 |
|  |  | Ni-Ni | 4.9 ± 0.4 | 3.10 ± 0.01 |  |  |  |
|  | TA-CNT-Ni-1.4V | Ni-O | 3.6 ± 0.4 | 1.90 ± 0.01 | 0.0028 ± 0.0010 | 8345.5 ± 0.7 | 0.0034 |
|  |  | Ni-O | 1.5 ± 0.2 | 2.08 ± 0.02 |  |  |  |
|  |  | Ni-Ni | 4.3 ± 0.5 | 2.84 ± 0.01 | 0.0054 ± 0.0021 |  |  |
|  |  | Ni-Ni | 2.1 ± 0.4 | 3.10 ± 0.01 |  |  |  |
|  | TA-CNT-Ni-1.6V | Ni-O | 5.4 ± 0.3 | 1.89 ± 0.01 | 0.0045 ± 0.0008 | 8344.5 ± 0.8 | 0.0068 |
|  |  | Ni-Ni | 5.1 ± 0.6 | 2.82 ± 0.01 |  |  |  |
|  | TA-CNT-FeNi-OCP | Ni-O | 5.9 ± 0.3 | 2.06 ± 0.01 | 0.0064 ± 0.0008 | 8341.4 ± 0.6 | 0.0061 |
|  |  | Ni-Fe/Ni | 5.9 ± 0.6 | 3.09 ± 0.01 |  |  |  |
|  | TA-CNT-FeNi-1.1V | Ni-O | 6.0 ± 0.4 | 2.06 ± 0.01 | 0.0067 ± 0.0011 | 8341.4 ± 0.6 | 0.0056 |
|  |  | Ni-Fe/Ni | 6.2 ± 0.7 | 3.09 ± 0.01 |  |  |  |
|  | TA-CNT-FeNi-1.4V | Ni-O | 5.8 ± 0.3 | 2.06 ± 0.01 | 0.0070 ± 0.0008 | 8341.3 ± 0.6 | 0.0058 |
|  |  | Ni-Fe/Ni | 6.0 ± 0.6 | 3.09 ± 0.01 |  |  |  |
|  | TA-CNT-FeNi-1.6V | Ni-O | 5.6 ± 0.4 | 1.89 ± 0.01 | 0.0037 ± 0.0008 | 8344.0 ± 0.9 | 0.0058 |
|  |  | Ni-Fe/Ni | 4.9 ± 0.6 | 2.82 ± 0.01 |  |  |  |

**Table S4.** Literature survey of Fe–O bond lengths at octahedrally coordinated Fe sites and Ni. Bond lengths 𝑑̅ are averaged from their contributions d where the local symmetry deviates from a perfect octahedron. XRD: X-ray diffraction, ND: neutron diffraction.

| **Compound** | **Oxidation**  **state** | **𝒅̅ (Å)** | **Method** | **Reference** |
| --- | --- | --- | --- | --- |
| **FeO** | +2 | 2.154 | XRD | *Acta Crystallogr.* 1953, 6, 827. |
| **FeO** | +2 | 2.150 | XRD | *Acta Crystallogr.* B 1982, 38, 1451. |
| **α-Fe_2_O_3_** | +3 | 2.031 | XRD | *Am. Mineral.* 1966, 51, 123. |
| **Fe_3_O_4_** | +3 | 2.058 | XRD | *Acta Crystallogr.* B 1981, 37, 917. |
| **α-FeOOH** | +3 | 2.018 | EXAFS | *Am. Mineral.* 2004, 89, 572. |
| **α-FeOOH** | +3 | 1.997 | XRD | *Phys. Status Solidi B* 1968, 26, 429. |
| **γ-FeOOH** | +3 | 2.041 | XRD | *Acta Chem. Scand. Ser. -Phys. Inorg. Chem.* 1978, 32, 87. |
| **γ-FeOOH** | +3 | 2.028 | ND | *Phys. Status Solidi B 1970, 41, 173.* |
| **α-Ni(OH)_2_** | +2 | 2.050 | EXAFS | *Appl. Phys. Lett.* 1991, 58, 865. |
| **α-Ni(OH)_2_** | +2 | 2.050 | EXAFS | *J. Phys. Chem. 1990, 94, 21.* |
| **β-Ni(OH)_2_** | +2 | 2.070 | EXAFS | *Appl. Phys. Lett.* 1991, 58, 865. |
| **β-Ni(OH)_2_** | +2 | 2.060 | EXAFS | *Phys. B Condens. Matter* 1995, 208–209, 583. |
| **β-Ni(OH)_2_** | +2 | 2.070 | EXAFS | *J. Electrochem. Soc.* 1990, 137, 383. |
| **β-NiOOH** | +3 | 1.959 | EXAFS | *A. Phys. B Condens.* Matter 1995, 208–209, 583. |
| **β-NiOOH** | +3 | 1.923 | EXAFS | *Nucl. Instrum. Methods Phys.*  *Res. Sect. B Beam Interact. Mater. At.* 1997, 133, 39. |
| **β-NiOOH** | +3 | 1.916 | EXAFS | *Electrochemistry* 2008, 76, 802. |
| **γ-NiOOH** | +3.67 | 1.880 | EAXFS | *J. Am. Chem. Soc.* 2012, 134, 6801. |
| **γ-NiOOH** | +3.67 | 1.900 | XRD | *J. Am. Chem. Soc.* 2012, 134, 6801. |
| **γ-NiOOH** | +3.67 | 1.888 | EAXFS | *Electrochemistry 2008, 76, 802.* |
| **γ-NiOOH** | +3.67 | 1.860 | EAXFS | *Appl. Phys. Lett.* 1991, 58, 865. |
| **KNiIO_6_** | +4 | 1.870 | EAXFS | *Electrochimica Acta* 2001, 46, 3119. |
| **KNiIO_6_** | +4 | 1.880 | EAXFS | *Phys. B Condens. Matter* 1995, 208–209, 583. |
| **KNiIO_6_** | +4 | 1.870 | EAXFS | *J. Chem. Soc. Dalton Trans.* 1994, 1483. |

**Table S5.** Cathodic redox peak fitting results for TA-CNT-Ni.

| **Cycle** | **(E_p) / V vs. Hg/HgO** | **(E_p) / V vs. RHE** | **Peak height / mA** | **Charge / mC** |
| --- | --- | --- | --- | --- |
| 1 | 0.3795 | 1.3049 | 17.55 | 15.45 |
| 5 | 0.3438 | 1.2692 | 22.87 | 17.91 |
| 10 | 0.3333 | 1.2587 | 23.74 | 17.88 |
| 15 | 0.3285 | 1.2539 | 24.06 | 17.85 |
| 20 | 0.3257 | 1.2511 | 24.26 | 17.86 |

**Table S6.** Comparison of OER performance and stability of the as-prepared catalysts with recent representative SACs.

| **Name** | **Overpotential**  **(mV)*** | **Stability**  **(h)** | **Loading mass**  **(mg cm^−2^)** | **Reference** |
| --- | --- | --- | --- | --- |
| **Co_SAC_-W*_x_*C** | 371 | - | 0.38 | *J. Am. Chem. Soc.* 146, 9124−9133 (**2024**) |
| **Carbon based Co SAC** | 351 | 300 | 0.205 | *J. Am. Chem. Soc.* 145, 8052−8063(**2023**) |
| **Ir SAC on NiO** | 256 | 20 | - | *J. Am. Chem. Soc*. 146, 32953−32964(**2024**) |
| **Co_SA_-MoCeO_x_@BCT** | 239 | 60 | - | *Energy Environ. Sci*. 17, 3088-3098(**2024**) |
| **Co_SA_-MoCeO_x_** | 333 | - | - | *Energy Environ. Sci*. 17, 3088-3098(**2024**) |
| **Ni−N−C** | ~370 | 0.83 | - | *Angew. Chem. Int. Ed.* 64, e202413308(**2025**) |
| **Co/Fe-SNC800** | 240 | 28 | - | *Energy Environ. Sci.*16, 1685-1696 (**2024**) |
| **Fe_1_(OH)*_x_*/P-C** | 320 | 20 | 0.85 | *Nano Lett.* 21, 4795−4801(**2021**) |
| **Fe-N-C/Fe_2_C-op** | 349 | - | - | *https://doi.org/10.1002/advs.202301656* |
| **Tan-CN-CoFe** | 370 | 60 | 0.13 | This work |
| **Tan-CN-NiFe** | 320 | 47.6 | 0.13 | This work |

**Table S7.** ICP-MS measurements of the electrolyte for all catalysts before and after OER.

|  | **Fe (ppm)** | **Co (ppm)** | **Ni (ppm)** |
| --- | --- | --- | --- |
| **Commercial 1M KOH** | < 0.7 | < 0.3 | 3.4 |
| **TA-CNT-Fe** | < 0.8 | < 1.0 | 3.5 |
| **TA-CNT-Ni** | < 0.9 | < 0.45 | 4.9 |
| **TA-CNT-NiFe** | < 0.8 | 0.36 | 4.6 |
| **TA-CNT-CoFe** | < 0.7 | 0.35 | 3.2 |

**Table S8**. Crystal structure parameters for Fe, Co and Ni reference compounds (data retrieved from ICSD).

| **Composition** | **Space**  **Group** | **Crystal**  **System** | **Scattering**  **paths** | **Coordination**  **numbers** | **Interatomic**  **Distances** | **ICSD**  **Index** | **PDF**  **number** | **Ref.** |
| --- | --- | --- | --- | --- | --- | --- | --- | --- |
| α-Fe | $Im\bar{3}m$ | Cubic | Fe-Fe | 8 | 2.48 Å | 52258 | 01-071-3763 | ^17^ |
|  |  |  | Fe-Fe | 6 | 2.87 Å |  |  |  |
| FeO | $Fm\bar{3}m$ | Cubic | Fe-O | 6 | 2.06 | 82233 | 01-089-0687 | ^18^ |
|  |  |  | Fe-Fe | 12 | 3.06 |  |  |  |
| α-Fe_2_O_3_ | $R\bar{3}c$ | Trigonal | Fe-O | 3 | 1.94 | 40142 | 01-086-2368 | ^19^ |
|  |  |  | Fe-O | 3 | 2.11 |  |  |  |
|  |  |  | Fe-Fe | 1 | 2.89 |  |  |  |
|  |  |  | Fe-Fe | 3 | 2.97 |  |  |  |
|  |  |  | Fe-Fe | 3 | 3.36 |  |  |  |
| Fe(OH)_2_ | $P\bar{3}m1$ | Trigonal | Fe-O | 6 | 2.23 | 53992 | 01-073-6991 | ^20^ |
|  |  |  | Fe-Fe | 6 | 3.25 |  |  |  |
| Fe(OH)_3_ *x*H_2_O | $Immm$ | Orthorhombic | Fe-O(1) | 2 | 1.99 | 73441 | 01-081-2022 | ^21^ |
|  |  |  | Fe-O(2) | 2 | 2.01 |  |  |  |
|  |  |  | Fe-O(3) | 2 | 2.03 |  |  |  |
|  |  |  | Fe-Fe | 2 | 3.77 |  |  |  |
|  |  |  | Fe-Fe | 4 | 3.78 |  |  |  |
| α-FeO(OH) | $Pbnm$ | Orthorhombic | Fe-O(1) | 2 | 1.94 | 239324 | 01-084-8280 | ^22^ |
|  |  |  | Fe-O(1) | 1 | 2.01 |  |  |  |
|  |  |  | Fe-O(2) | 1 | 2.07 |  |  |  |
|  |  |  | Fe-O(2) | 2 | 2.11 |  |  |  |
|  |  |  | Fe-Fe | 2 | 3.02 |  |  |  |
|  |  |  | Fe-Fe | 2 | 3.21 |  |  |  |
|  |  |  | Fe-Fe | 4 | 3.52 |  |  |  |
| δ-FeO(OH) | $P\bar{3}m1$ | Trigonal | Fe(1)-O | 6 | 2.04 | 38299 | 01-077-0247 | ^23^ |
|  |  |  | Fe(2)-O | 6 | 2.06 |  |  |  |
|  |  |  | Fe(1)-Fe(1) | 3 | 2.95 |  |  |  |
|  |  |  | Fe(2)-Fe(2) | 3 | 2.95 |  |  |  |
| β-FeO(OH) | $I4/m$ | Tetragonal | Fe-O(1) | 2 | 2.01 | 31136 | 01-075-1594 | ^24^ |
|  |  |  | Fe-O(1) | 1 | 2.03 |  |  |  |
|  |  |  | Fe-O(2) | 1 | 2.05 |  |  |  |
|  |  |  | Fe-O(2) | 2 | 2.20 |  |  |  |
|  |  |  | Fe-Fe | 2 | 3.02 |  |  |  |
|  |  |  | Fe-Fe | 2 | 3.09 |  |  |  |
| γ-FeO(OH) | $Cmcm$ | Orthorhombic | Fe-O(1) | 2 | 1.97 | 24885 | 01-073-2326 | ^25^ |
|  |  |  | Fe-O(2) | 2 | 1.98 |  |  |  |
|  |  |  | Fe-O(1) | 2 | 2.13 |  |  |  |
|  |  |  | Fe-Fe | 2 | 3.06 |  |  |  |
|  |  |  | Fe-Fe | 4 | 3.10 |  |  |  |
| Co | $Fm\bar{3}m$ | Cubic | Co-Co | 12 | 2.51 Å | 76632 | 01-089-7093 | ^26^ |
| Co | $P6_{3}/mmc$ | Cubic | Co-Co | 6 | 2.50 Å | 76633 | 01-089-7094 | ^26^ |
|  |  |  | Co-Co | 6 | 2.51 Å |  |  |  |
| CoO | $Fm\bar{3}m$ | Cubic | Co-O | 6 | 2.13 Å | 9865 | 01-071-1178 | ^27^ |
|  |  |  | Co-Co | 12 | 3.01 Å |  |  |  |
| Co_3_O_4_ | $Fd\bar{3}m$ | Cubic | Co(1)-O | 6 | 1.91 Å | 36256 | 01-076-1802 | ^28^ |
|  |  |  | Co(2)-O | 4 | 1.94 Å |  |  |  |
|  |  |  | Co(1)-Co(1) | 6 | 2.85 Å |  |  |  |
|  |  |  | Co(1)-Co(2) | 6 | 3.35 Å |  |  |  |
|  |  |  | Co(2)-Co(1) | 12 | 3.35 Å |  |  |  |
|  |  |  | Co(2)-Co(2) | 4 | 3.50 Å |  |  |  |
| β-Co(OH)_2_ | $P\bar{3}m1$ | Trigonal | Co-O | 6 | 2.12 Å | 88940 | 01-089-8616 | ^29^ |
|  |  |  | Co-Co | 6 | 3.19 Å |  |  |  |
| CoO(OH) | $R\bar{3}m$ | Trigonal | Co-O | 6 | 1.90 Å | 22285 | 01-073-0497 | ^30^ |
|  |  |  | Co-Co | 6 | 2.85 Å |  |  |  |
| Ni | $Fm\bar{3}m$ | Cubic | Ni-Ni | 12 | 2.49 Å | 52231 | 01-071-3740 | ^31^ |
| NiO | $Fm\bar{3}m$ | Cubic | Ni-O | 6 | 2.09 Å | 9866 | 01-071-1179 | ^27^ |
|  |  |  | Ni-Ni | 12 | 2.95 Å |  |  |  |
| β-Ni(OH)_2_ | $P\bar{3}m1$ | Trigonal | Ni-O | 6 | 2.06 Å | 169978 | 01-080-2855 | ^32^ |
|  |  |  | Ni-Ni | 6 | 3.10 Å |  |  |  |
| NiO(OH) | $C2/m$ | Monoclinic | Ni-O(1) | 3 | 1.91 Å | 230448 | 01-086-8225 | ^33^ |
|  |  |  | Ni-O(2) | 1 | 1.98 Å |  |  |  |
|  |  |  | Ni-O(2) | 2 | 2.12 Å |  |  |  |
|  |  |  | Ni-Ni | 4 | 2.84 Å |  |  |  |
|  |  |  | Ni-Ni | 2 | 2.91 Å |  |  |  |

**Figure 9.** Crystal structure parameters for Fe, Co and Ni reference compounds (data retrieved from ICSD).

| **Name** | **(h, k, l)** | **2** **θ** | **d (Å)** | **Intensity** |
| --- | --- | --- | --- | --- |
| **β-NiOOH**  **Space group:** [C 1 2/m 1 (12)](https://icsd.fiz-karlsruhe.de/linkicsd.xhtml?link_back=true&expert_query=spacegroupsymbolexact%3A+%22C12%2Fm1%22&link_name=C12%2Fm1&link_label=All+structures+of+space+group+symbol)  **PDF:** 01-078-4225  **Ref.:** [Materials Research Society Symposium Proceedings](https://icsd.fiz-karlsruhe.de/linkicsd.xhtml?link_back=true&expert_query=journal%3A+Materials+Research+Society+Symposium+Proceedings&link_name=Materials+Research+Society+Symposium+Proceedings&link_label=All+entries+like+journal+title) (2009) [1126](https://icsd.fiz-karlsruhe.de/linkicsd.xhtml?link_back=true&expert_query=volume%3A1126&link_name=1126&link_label=All+ICSD+entries+of+a+journal+volume), p. 131-136 |  |  |  |  |
|  | 001 | 9.57 | 9.238 | 17.2 |
|  | 002 | 19.20 | 4.6190 | 1000 |
|  | 003 | 28.97 | 3.0793 | 9.8 |
|  | 110 | 35.80 | 2.5060 | 83.4 |
|  | 1-10 | 35.80 | 2.5060 | 83.4 |
|  | 200 | 36.79 | 2.4410 | 8.6 |
|  | 11-1 | 37.04 | 2.4251 | 147.6 |
|  | 1-1-1 | 37.04 | 2.4251 | 147.6 |
|  | 1-11 | 37.25 | 2.4120 | 22.8 |
|  | 111 | 37.25 | 2.4120 | 22.8 |
|  | 20-1 | 37.09 | 2.3723 | 48.0 |
|  | 201 | 38.31 | 2.3479 | 80.3 |
|  | 004 | 38.97 | 2.3095 | 39.2 |
|  | 11-2 | 40.75 | 2.2127 | 106.6 |
|  | 1-1-2 | 40.75 | 2.2127 | 106.6 |
|  | 112 | 41.13 | 2.1928 | 12.1 |
|  | 1-12 | 41.13 | 2.1928 | 12.1 |
|  | 20-2 | 41.44 | 2.1771 | 3.6 |
|  | 202 | 42.20 | 2.1397 | 0.1 |
| **γ-NiOOH**    Space group: C1m1  **ICSD Entry: 169981**  Solid State Ionics, 2010, 181, 1764, DOI: [10.1016/j.ssi.2010.10.002](http://dx.doi.org/10.1016/j.ssi.2010.10.002) | 00-1 | 20.14 | 4.40 | 1000 |
|  | 001 | 20.14 | 4.40 | 99.9 |
|  | -200 | 34.58 | 2.59 | 30.36 |
|  | 200 | 34.58 | 2.59 | 30.36 |
|  | -201 | 35.51 | 2.52 | 3.72 |
|  | 20-1 | 35.51 | 2.52 | 3.72 |
|  | -110 | 39.21 | 2.2 | 48.8 |
|  | 110 | 39.21 | 2.29 | 48.8 |
|  | 00-2 | 40.94 | 2.20 | 2.8 |
|  | 002 | 40.94 | 2.20 | 2.8 |
|  | 111 | 43.70 | 2.06 | 7.8 |
|  | -11-1 | 43.70 | 2.06 | 7.8 |
|  | -20-1 | 44.75 | 2.02 | 57.3 |
|  | 201 | 44.75 | 2.02 | 57.3 |
| **β-Ni(OH)_2_**  **Space group: P -3 m 1 (164)**  **PDF:** 01-076-8985  **Ref.:** Materials Research Bulletin (2008) 43, p. 3227-3233 |  |  |  |  |
|  | 001 | 19.22 | 4.6147 | 1000 |
|  | 1-10 | 33.02 | 2.7103 | 107.4 |
|  | 100 | 33.02 | 2.7103 | 107.4 |
|  | 010 | 33.02 | 2.7103 | 107.4 |
|  | 01-1 | 38.49 | 2.337 | 34.7 |
|  | 10-1 | 38.49 | 2.337 | 234.6 |
|  | 1-11 | 38.49 | 2.337 | 234.6 |
|  | 1-1-1 | 38.49 | 2.337 | 34.7 |
|  | 011 | 38.49 | 2.337 | 234.6 |
|  | 101 | 38.49 | 2.337 | 34.7 |
|  | 002 | 39 | 2.3073 | 35.3 |
| **β-FeOOH**  **Space Group: I 4/m**  **ICSD Entry: 167358**  **A. Szytula, M. Balanda, Z. Dimitrijevic, Physica Status Solidi, A, 1970, 8, 1033, DOI: 10.1002/pssa.19700030421** | 110 | 11.88 | 7.43 | 100 |
|  | 200 | 16.84 | 5.25 | 0 |
|  | 220 | 23.90 | 3.71 | 0 |
|  | 030 | 25.38 | 3.50 | 0 |
|  | 310 | 26.77 | 3.32 | 43.24 |
|  | 001 | 28.85 | 3.09 | 0 |
|  | 101 | 30.1 | 2.96 | 0 |
|  | 011 | 30.1 | 2.96 | 0 |
|  | 040 | 34.0 | 2.62 | 33.38 |
|  | -211 | 34.69 | 2.58 | 22.55 |
| **γ-FeOOH**  **Space Group:** C m c m  ICSD Entry: 24885  A. Oles, A. Szytula, A. Wanic, Physica Status Solidi, 1970, 41, 173, DOI: [10.1002/pssb.19700410119](http://dx.doi.org/10.1002/pssb.19700410119) | 200 | 14.7 | 6.0 | 100 |
|  | 300 | 22.19 | 4.0 | 0 |
|  | -201 | 27.16 | 3.28 | 52.88 |
|  | 310 | 36.67 | 2.44 | 53.39 |
|  | 002 | 46.53 | 1.95 | 33.67 |

**Table S10.** Raman shift assignments for TA-CNT-NiFe, TA-CNT-Ni, TA-CNT-Fe as well as corresponding references.

| **Raman Shift (cm⁻¹)** | **Possible Assignment** | **References** |
| --- | --- | --- |
| ~580 | M–O stretching in FeOOH or NiFeOOH | Applied Catalysis B: Environmental 318 (2022) 121825 |
| 630–685 | M–O stretching (Ni–O/Fe–O) or –OH bending; *OH absorbed on the Ni site is 685 cm^-1^; the strong peak at ∼675 cm^–1^ is attributed to the characteristic octahedral-site A_1g_ | Applied Catalysis B: Environmental 318 (2022) 121825; Journal of Electroanalytical Chemistry 871 (2020) 114282; Chem. Sci., 2022, 13, 5639–5649; ACS Catal. 2023, 13, 14975−14986; *Chem. Mater.* 2020, 32, 10, 4303–4311 |
| 1020–1030 | *OOH intermediate (δ–O–O) stretching mode* | J. Am. Chem. Soc. 2015, 137, 15112−15121; Angew. Chem. Int. Ed. 2019, 58, 1252 – 1265; Angew. Chem. Int. Ed. 2021, 60, 19774 – 19778 |
| 1059 ±2 | High-valent Ni⁴⁺–O vibration | J. Am. Chem. Soc. 2015, 137, 15112−15121; Angew. Chem. Int. Ed. 2021, 60, 19774 – 19778 |
| 1358 | D band (defect mode in carbon) | Arises from sp^3^ carbon or oxygen functional groups on CNT |
| 1590 | G band (graphitic carbon mode) | In-plane E_2_g vibration of sp^2^ carbon networks |
| 1620 | D′ band (disordered/edge carbon) | Often linked to edge defects or functionalized carbon domains |
| 2697 | 2D band (second-order graphitic mode) | Sensitive to graphitic layer stacking and structural order |
| 2947 | G + D combination overtone (carbon) | Second-order band from simultaneous G and D scattering, reflects carbon disorder and hybrid zones |

# References

(1)	Francisco de la Peña; Eric Prestat; Jonas Lähnemann; Vidar Tonaas Fauske; Pierre Burdet; Petras Jokubauskas; Tom Furnival; Carter Francis; Magnus Nord; Tomas Ostasevicius; Katherine E. MacArthur; Duncan N. Johnstone; Mike Sarahan; Joshua Taillon; Thomas Aarholt; pquinn-dls; Vadim Migunov; Alberto Eljarrat; Jan Caron; T. Nemoto; Timothy Poon; Stefano Mazzucco; sivborg; actions-user; Nicolas Tappy; Niels Cautaerts; Suhas Somnath; Tom Slater; Michael Walls; pietsjoh. Hyperspy/Hyperspy: V2.3.0, 2025. https://doi.org/10.5281/ZENODO.592838.

(2)	van der Walt, S.; Schönberger, J. L.; Nunez-Iglesias, J.; Boulogne, F.; Warner, J. D.; Yager, N.; Gouillart, E.; Yu, T. Scikit-Image: Image Processing in Python. *PeerJ* **2014**, *2*, e453. https://doi.org/10.7717/peerj.453.

(3)	Virtanen, P.; Gommers, R.; Oliphant, T. E.; Haberland, M.; Reddy, T.; Cournapeau, D.; Burovski, E.; Peterson, P.; Weckesser, W.; Bright, J.; Van Der Walt, S. J.; Brett, M.; Wilson, J.; Millman, K. J.; Mayorov, N.; Nelson, A. R. J.; Jones, E.; Kern, R.; Larson, E.; Carey, C. J.; Polat, İ.; Feng, Y.; Moore, E. W.; VanderPlas, J.; Laxalde, D.; Perktold, J.; Cimrman, R.; Henriksen, I.; Quintero, E. A.; Harris, C. R.; Archibald, A. M.; Ribeiro, A. H.; Pedregosa, F.; Van Mulbregt, P.; SciPy 1.0 Contributors; Vijaykumar, A.; Bardelli, A. P.; Rothberg, A.; Hilboll, A.; Kloeckner, A.; Scopatz, A.; Lee, A.; Rokem, A.; Woods, C. N.; Fulton, C.; Masson, C.; Häggström, C.; Fitzgerald, C.; Nicholson, D. A.; Hagen, D. R.; Pasechnik, D. V.; Olivetti, E.; Martin, E.; Wieser, E.; Silva, F.; Lenders, F.; Wilhelm, F.; Young, G.; Price, G. A.; Ingold, G.-L.; Allen, G. E.; Lee, G. R.; Audren, H.; Probst, I.; Dietrich, J. P.; Silterra, J.; Webber, J. T.; Slavič, J.; Nothman, J.; Buchner, J.; Kulick, J.; Schönberger, J. L.; De Miranda Cardoso, J. V.; Reimer, J.; Harrington, J.; Rodríguez, J. L. C.; Nunez-Iglesias, J.; Kuczynski, J.; Tritz, K.; Thoma, M.; Newville, M.; Kümmerer, M.; Bolingbroke, M.; Tartre, M.; Pak, M.; Smith, N. J.; Nowaczyk, N.; Shebanov, N.; Pavlyk, O.; Brodtkorb, P. A.; Lee, P.; McGibbon, R. T.; Feldbauer, R.; Lewis, S.; Tygier, S.; Sievert, S.; Vigna, S.; Peterson, S.; More, S.; Pudlik, T.; Oshima, T.; Pingel, T. J.; Robitaille, T. P.; Spura, T.; Jones, T. R.; Cera, T.; Leslie, T.; Zito, T.; Krauss, T.; Upadhyay, U.; Halchenko, Y. O.; Vázquez-Baeza, Y. SciPy 1.0: Fundamental Algorithms for Scientific Computing in Python. *Nat Methods* **2020**, *17* (3), 261–272. https://doi.org/10.1038/s41592-019-0686-2.

(4)	Belin, S.; Briois, V.; Traverse, A.; Idir, M.; Moreno, T.; Ribbens, M. SAMBA a New Beamline at SOLEIL for XRay Absorption Spectroscopy in the 4–40keV Energy Range. *Physica Scripta* **2005**, 980. https://doi.org/10.1238/Physica.Topical.115a00980.

(5)	Briois, V.; Fonda, E.; Belin, S.; Barthe, L.; La Fontaine, C.; Langlois, F.; Ribbens, M.; Villain, F. SAMBA: The 4–40 keV X-Ray Absorption Spectroscopy Beamline at SOLEIL. In *UVX 2010 - 10e Colloque sur les Sources Cohérentes et Incohérentes UV, VUV et X ; Applications et Développements Récents*; EDP Sciences: Ile de Porquerolles, France, **2011**; pp 41–47. https://doi.org/10.1051/uvx/2011006.

(6)	Fonda, E.; Rochet, A.; Ribbens, M.; Barthe, L.; Belin, S.; Briois, V. The SAMBA Quick-EXAFS Monochromator: XAS with Edge Jumping. *J Synchrotron Rad* **2012**, *19* (3), 417–424. https://doi.org/10.1107/S0909049512009703.

(7)	Dent, A. J.; Cibin, G.; Ramos, S.; Smith, A. D.; Scott, S. M.; Varandas, L.; Pearson, M. R.; Krumpa, N. A.; Jones, C. P.; Robbins, P. E. B18: A Core XAS Spectroscopy Beamline for Diamond. *J. Phys.: Conf. Ser.* **2009**, *190*, 012039. https://doi.org/10.1088/1742-6596/190/1/012039.

(8)	Dent, A. J.; Cibin, G.; Ramos, S.; Parry, S. A.; Gianolio, D.; Smith, A. D.; Scott, S. M.; Varandas, L.; Patel, S.; Pearson, M. R.; Hudson, L.; Krumpa, N. A.; Marsch, A. S.; Robbins, P. E. Performance of B18, the Core EXAFS Bending Magnet Beamline at Diamond. *J. Phys.: Conf. Ser.* **2013**, *430*, 012023. https://doi.org/10.1088/1742-6596/430/1/012023.

(9)	Ravel, B.; Newville, M. *ATHENA* , *ARTEMIS* , *HEPHAESTUS* : Data Analysis for X-Ray Absorption Spectroscopy Using *IFEFFIT*. *J Synchrotron Rad* **2005**, *12* (4), 537–541. https://doi.org/10.1107/S0909049505012719.

(10)	Muñoz, M.; Argoul, P.; Farges, F. Continuous Cauchy Wavelet Transform Analyses of EXAFS Spectra: A Qualitative Approach. *American Mineralogist* **2003**, *88* (4), 694–700. https://doi.org/10.2138/am-2003-0423.

(11)	Newville, M. Larch: An Analysis Package for XAFS and Related Spectroscopies. *J. Phys.: Conf. Ser.* **2013**, *430*, 012007. https://doi.org/10.1088/1742-6596/430/1/012007.

(12)	Ofuchi, H.; Matsumoto, T.; Honma, T. Construction of XAFS Standard Sample Database at BL14B2 in SPring-8. *Radiation Physics and Chemistry* **2024**, *218*, 111581. https://doi.org/10.1016/j.radphyschem.2024.111581.

(13)	Blöchl, P. E. Projector Augmented-Wave Method. *Phys. Rev. B* **1994**, *50* (24), 17953–17979. https://doi.org/10.1103/PhysRevB.50.17953.

(14)	Perdew, J. P.; Burke, K.; Ernzerhof, M. Generalized Gradient Approximation Made Simple. *Phys. Rev. Lett.* **1996**, *77* (18), 3865–3868. https://doi.org/10.1103/PhysRevLett.77.3865.

(15)	Grimme, S. Semiempirical GGA-type density functional constructed with a long-range dispersion correction. *Journal of Computational Chemistry* **2006**, *27* (15), 1787–1799. https://doi.org/10.1002/jcc.20495.

(16)	Friebel, D.; Louie, M. W.; Bajdich, M.; Sanwald, K. E.; Cai, Y.; Wise, A. M.; Cheng, M.-J.; Sokaras, D.; Weng, T.-C.; Alonso-Mori, R.; Davis, R. C.; Bargar, J. R.; Nørskov, J. K.; Nilsson, A.; Bell, A. T. Identification of Highly Active Fe Sites in (Ni,Fe)OOH for Electrocatalytic Water Splitting. *J. Am. Chem. Soc.* **2015**, *137* (3), 1305–1313. https://doi.org/10.1021/ja511559d.

(17)	Owen, E. A.; Yates, E. L. XLI. *Precision Measurements of Crystal Parameters*. *The London, Edinburgh, and Dublin Philosophical Magazine and Journal of Science* **1933**, *15* (98), 472–488. https://doi.org/10.1080/14786443309462199.

(18)	Fjellvåg, H.; Grønvold, F.; Stølen, S.; Hauback, B. On the Crystallographic and Magnetic Structures of Nearly Stoichiometric Iron Monoxide. *Journal of Solid State Chemistry* **1996**, *124* (1), 52–57. https://doi.org/10.1006/jssc.1996.0206.

(19)	Maslen, E. N.; Streltsov, V. A.; Streltsova, N. R.; Ishizawa, N. Synchrotron X-Ray Study of the Electron Density in α-Fe_2_O_3_. *Acta Crystallogr B Struct Sci* **1994**, *50* (4), 435–441. https://doi.org/10.1107/S0108768194002284.

(20)	Lincei, A. nazionale dei; Classe di scienze fisiche, matematiche; naturali. *Atti Della R. Accademia Nazionale Dei Lincei. Memorie Della Classe Di Scienze Fisiche, Matematiche e Naturali*; R. Accademia nazionale dei Lincei, **1927**.

(21)	Birch, W. D.; Pring, A.; Reller, A.; Schmalle, H. W. Bernalite, Fe(OH)_3_, a New Mineral from Broken Hill, New South Wales: Description and Structure. *American Mineralogist* **1993**, *78* (7–8), 827–834.

(22)	Zepeda-Alarcon, E.; Nakotte, H.; Gualtieri, A. F.; King, G.; Page, K.; Vogel, S. C.; Wang, H.-W.; Wenk, H.-R. Magnetic and Nuclear Structure of Goethite (α-FeOOH): A Neutron Diffraction Study. *J Appl Crystallogr* **2014**, *47* (6), 1983–1991. https://doi.org/10.1107/S1600576714022651.

(23)	Patrat, G.; De Bergevin, F.; Pernet, M.; Joubert, J. C. Structure Locale de δ-FeOOH. *Acta Crystallogr B Struct Sci* **1983**, *39* (2), 165–170. https://doi.org/10.1107/S0108768183002232.

(24)	Mackay, A. L. β-Ferric Oxyhydroxide. *Mineral. mag. j. Mineral. Soc.* **1960**, *32* (250), 545–557. https://doi.org/10.1180/minmag.1960.032.250.04.

(25)	Oleś, A.; Szytuła, A.; Wanic, A. Neutron Diffraction Study of γ‐FeOOH. *Physica Status Solidi (b)* **1970**, *41* (1), 173–177. https://doi.org/10.1002/pssb.19700410119.

(26)	Taylor, A.; Floyd, R. W. Precision Measurements of Lattice Parameters of Non-Cubic Crystals. *Acta Cryst* **1950**, *3* (4), 285–289. https://doi.org/10.1107/S0365110X50000732.

(27)	Sasaki, S.; Fujino, K.; Takéuchi, Y. X-Ray Determination of Electron-Density Distributions in Oxides, MgO, MnO, CoO, and NiO, and Atomic Scattering Factors of Their Constituent Atoms. *Proc. Jpn. Acad., Ser. B* **1979**, *55* (2), 43–48. https://doi.org/10.2183/pjab.55.43.

(28)	Picard, J. P.; Baud, G.; Besse, J. P.; Chevalier, R. Croissance cristalline et étude structurale de Co_3_O_4_. *Journal of the Less Common Metals* **1980**, *75* (1), 99–104. https://doi.org/10.1016/0022-5088(80)90373-2.

(29)	Pertlik, F. The Distortion of the Hexagonal Close Packing of Oxygen Atoms in Co(OH)_2_ Compared to Isotypic Brucite-Type Structures. *Monatshefte fuer Chemie* **1999**, *130* (9), 1083–1088. https://doi.org/10.1007/PL00010286.

(30)	Delaplane, R. G.; Ibers, J. A.; Ferraro, J. R.; Rush, J. J. Diffraction and Spectroscopic Studies of the Cobaltic Acid System HCoC2–DCoO2. *The Journal of Chemical Physics* **1969**, *50* (5), 1920–1927. https://doi.org/10.1063/1.1671307.

(31)	Owen, E. A.; Yates, E. L. LXVI. *X-Ray Measurement of the Thermal Expansion of Pure Nickel*. *The London, Edinburgh, and Dublin Philosophical Magazine and Journal of Science* **1936**, *21* (142), 809–819. https://doi.org/10.1080/14786443608561628.

(32)	Kazimirov, V. Yu.; Smirnov, M. B.; Bourgeois, L.; Guerlou-Demourgues, L.; Servant, L.; Balagurov, A. M.; Natkaniec, I.; Khasanova, N. R.; Antipov, E. V. Atomic Structure and Lattice Dynamics of Ni and Mg Hydroxides. *Solid State Ionics* **2010**, *181* (39–40), 1764–1770. https://doi.org/10.1016/j.ssi.2010.10.002.

(33)	Casas-Cabanas, M.; Radin, M. D.; Kim, J.; Grey, C. P.; Van Der Ven, A.; Palacín, M. R. The Nickel Battery Positive Electrode Revisited: Stability and Structure of the β-NiOOH Phase. *J. Mater. Chem. A* **2018**, *6* (39), 19256–19265. https://doi.org/10.1039/C8TA07460G.
